# Antimicrobial Resistance: Its Surveillance, Impact, and Alternative Management Strategies in Dairy Animals

**DOI:** 10.3389/fvets.2017.00237

**Published:** 2018-01-08

**Authors:** Chetan Sharma, Namita Rokana, Mudit Chandra, Brij Pal Singh, Rohini Devidas Gulhane, Jatinder Paul Singh Gill, Pallab Ray, Anil Kumar Puniya, Harsh Panwar

**Affiliations:** ^1^Department of Dairy Microbiology, College of Dairy Science and Technology, Guru Angad Dev Veterinary and Animal Sciences University (GADVASU), Ludhiana, India; ^2^Department of Veterinary Microbiology, College of Veterinary Science, Guru Angad Dev Veterinary and Animal Sciences University (GADVASU), Ludhiana, India; ^3^School of Public Health and Zoonoses, Guru Angad Dev Veterinary and Animal Sciences University (GADVASU), Ludhiana, India; ^4^Department of Medical Microbiology, Post Graduate Institute for Medical Education and Research (PGIMER), Chandigarh, India

**Keywords:** antimicrobial resistance, antimicrobial usage, multidrug resistance, dairy farming, surveillance, alternative treatment strategies

## Abstract

Antimicrobial resistance (AMR), one among the most common priority areas identified by both national and international agencies, is mushrooming as a silent pandemic. The advancement in public health care through introduction of antibiotics against infectious agents is now being threatened by global development of multidrug-resistant strains. These strains are product of both continuous evolution and un-checked antimicrobial usage (AMU). Though antibiotic application in livestock has largely contributed toward health and productivity, it has also played significant role in evolution of resistant strains. Although, a significant emphasis has been given to AMR in humans, trends in animals, on other hand, are not much emphasized. Dairy farming involves surplus use of antibiotics as prophylactic and growth promoting agents. This non-therapeutic application of antibiotics, their dosage, and withdrawal period needs to be re-evaluated and rationally defined. A dairy animal also poses a serious risk of transmission of resistant strains to humans and environment. Outlining the scope of the problem is necessary for formulating and monitoring an active response to AMR. Effective and commendably connected surveillance programs at multidisciplinary level can contribute to better understand and minimize the emergence of resistance. Besides, it requires a renewed emphasis on investments into research for finding alternate, safe, cost effective, and innovative strategies, parallel to discovery of new antibiotics. Nevertheless, numerous direct or indirect novel approaches based on host–microbial interaction and molecular mechanisms of pathogens are also being developed and corroborated by researchers to combat the threat of resistance. This review places a concerted effort to club the current outline of AMU and AMR in dairy animals; ongoing global surveillance and monitoring programs; its impact at animal human interface; and strategies for combating resistance with an extensive overview on possible alternates to current day antibiotics that could be implemented in livestock sector.

## Introduction

Microorganisms are among the man’s best friends and also worst enemies. Knowledge about them has grown at fast pace; since their discovery by Leeuwenhoek and other eminent scientists, recognizing them as agents of infection. It took time to establish their role in food and fermentation and later their positive impact over human health (1). Exploitation of microbes and their metabolites for their useful applications in food, feed, dairy, fermentation, pharmaceutical, and other areas is practiced since centuries now (2–4). However, besides having beneficial roles, their impact as a threatening agent against humans, animals, and vegetation persists in form of many infections in human and animals and food spoilage, adding considerable load on individual and global economy. To counter these living threat agents, several measures, especially administration of antimicrobials, are employed globally. Discovery of first antibiotic, penicillin, retarded the prevalence of infectious diseases and saved millions of life particularly during Second World War. However, Sir Alexander Fleming, during his Nobel Prize speech in 1945, stated that bacteria could develop resistance against antibacterial therapies, and it was not much later when cases of non-efficacy of the wonder drug itself were reported (5, 6). This started the reporting of failure of other therapeutic drugs against infectious agents, later recognized as drug resistance, antimicrobial resistance (AMR), or antibiotic resistance; challenging the efficacy of modern therapeutic regimes. AMR in microbes is defined as their unresponsiveness to standard doses of clinically relevant antimicrobial drugs (7). Broadly, it is the property of microbes that overpower the antagonistic effects of antibiotics, to which they were earlier sensitive, resulting in their survival despite exposure to standard doses of antibiotic. This natural phenomenon further gets accelerated by the selective pressure generated by the use; more correctly, misuse of antibiotics. AMR has emerged as a threat to the current effective treatment for an ever-increasing range of microbial infections. It results in reduced efficacy of antibiotics; making treatment complicated, time consuming, costly, or sometimes even impossible. The discorvery of each and every new antibiotic has been followed by reports of emerging resistance against it (6).

**Figure 1:**
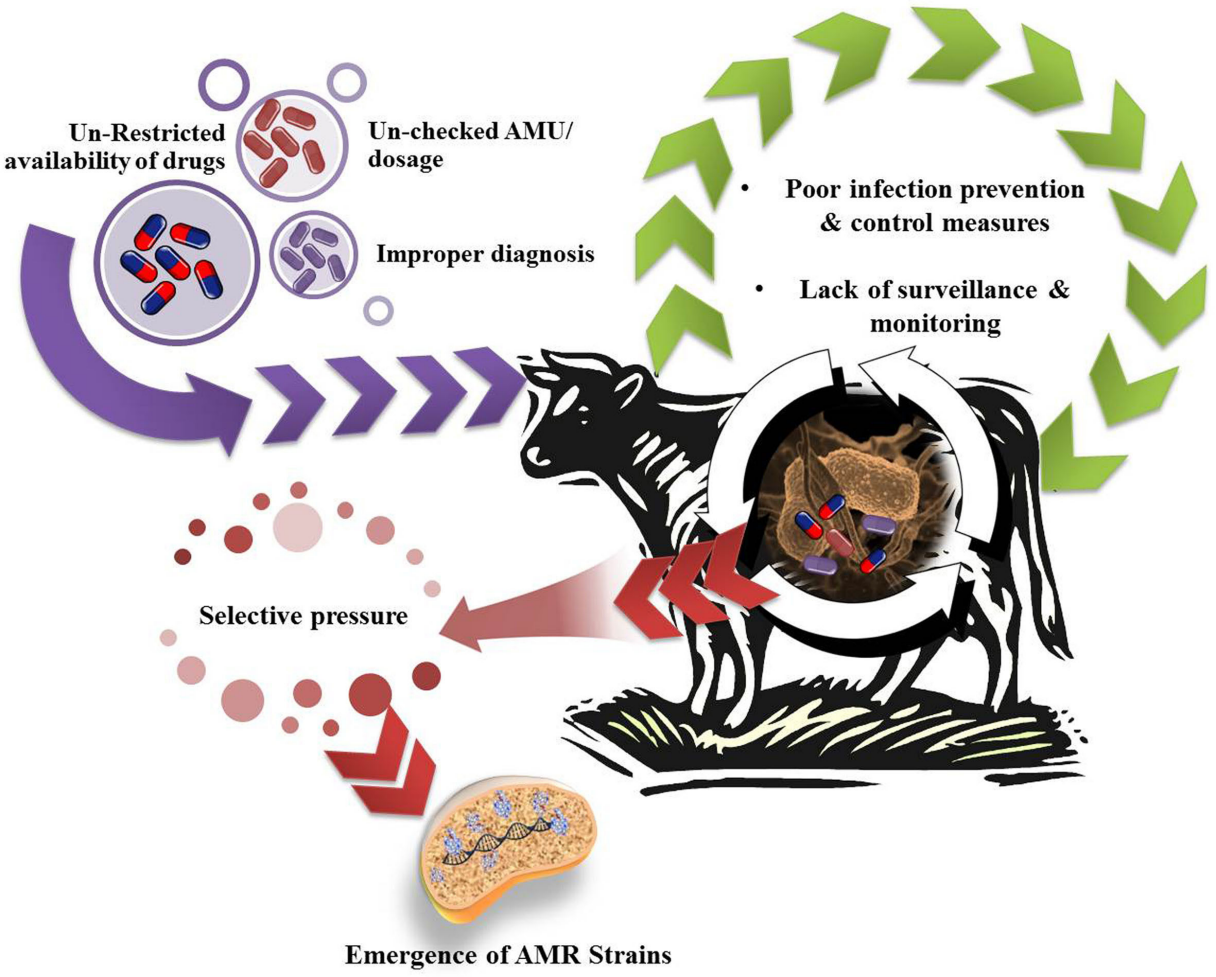
Possible factors leading to antimicrobial resistance (AMR) in dairy animals.

Further, AMR do not respect geographical boundaries and can traverse among humans, animals across countries, mediated through resistant strains; without any specific information and check (8). In the era of globalization and urbanization, prescribed treatment fails to put a check over resistant strains; infectious diseases become uncontrollable; major surgeries are jeopardized; and ultimately resistant forms are left free to spread (9). Different countries and research bodies are reacting toward issue of AMR and millions of dollars are being spent over surveillance and research for the abatement of AMR in human pathogens. AMR in humans has been inter-connected with AMR in other populations and ecosystems. The problem of AMR is equally important and prevalent in animals; although emphasized to a lower extent. Antimicrobial agents are being employed for food animal production either as therapeutic, metaphylactic, prophylactic, or as growth promoter (10). As an outcome of extensive public health concern regarding antimicrobial growth promoter (AGP) usage in livestock, the European Union (EU) progressively banned all AGPs in the livestock industry (11). The emergence and spread of drug-resistant bacteria arise from a myriad of ecological and evolutionary interacting factors, either natural or human-driven. Widespread dependence over antimicrobial usage (AMU) in animals results in a selective pressure under which bacteria can either develop resistance-mediating mutations or acquire resistance genes. Indeed, the usage of antimicrobial agents is perhaps the major driving force in resistance development and dissemination (12). Possible factors associated with AMR in animal microflora have been depicted in Figure 1. The rapid emergence of resistance toward current day antibiotics generates a potential scope for modern and novel antibiotics for futuristic approaches (13). AMR is affecting diverse populations globally and requires a cheap and effective treatment/prevention strategy for public well-being (14). Hence, there is an urgent need to survey and study the prevalence of AMR in commensal and pathogenic flora of animals, especially of economic value, i.e., milking herds, to understand the possible mechanism of AMR and development of natural treatment strategies with novel target sites, which can prove to be alternative strategies for combating AMR. So, sufficiently detailed knowledge about multidrug-resistant (MDR) bacteria (quantitative understanding of the dynamics), and multiple resistance determinants in variable host (humans and animals) and different environmental compartments, is required to make useful predictions and designing regulatory measures at national as well as international levels. In light of emerging aspect of drug resistance, the present review underlines the responsible factors for developing burden of AMR crisis in dairy cattle. The role of concerned regulating authorities and practitioners for implementation of policies for antibiotic stewardship to seize the rising threats is also featured. Furthermore, we also record the plausible alternative therapeutic strategies currently being used or studied to limit AMR problem.

## AMU and Resistance in Dairy Animals

Milk, being the most popular natural health food, is consumed globally by members of every age group. This forms the basis of establishment of dairy farming and industry. Besides milk, dairy animals are also reared for meat purpose. Dairy animals maintained in large groups and conventional dairy farms are more frequently exposed to antimicrobials in comparison to those maintained as small holdings and practicing organic farming. The demand for animal source food is also increasing at a rapid rate (15). With this increasing demand, the value of veterinary drugs in international market augmented from 8.65 billion dollar in 1992 to 20 billion dollar in 2010 and is projected to touch 42.9 billion dollar mark by 2018 (16). Globally, animal farming relies heavily on the surplus use of antimicrobials for the improvement of animal health and greater productivity. According to Food and Drug Administration (FDA) (17), AMU in food animals in USA was estimated to account for 80% of the total nation’s annual antimicrobial consumption. In 2012, 26 EU countries’ average consumption of antimicrobials was 116.4 and 144.0 mg/kg of estimated biomass in humans and animals, respectively (18). Increasing population will further generate a demand for high-quality livestock products. In order to match this global demand for animal proteins, AMU in food animal production will rise over 67% by 2030 (19). The episodes of widespread resistance may be more consequential for developing countries, including India, where the infectious disease burden is very high and, therefore, attracts higher antimicrobial application for limiting morbidity and mortality (7). Presence of antimicrobial residues in food animal products (chicken meat and milk) has been reported from different parts of India, indicating wide AMU in food animal production in India (20–23). Predicting actual values of AMU in dairy farms is challenged by several factors, *viz*., lack of maintenance of antibiotic treatment records and written plans for treating sick animals; low dependence on veterinarian’s advice; and administration by the owner itself. Also, there is considerable variation in the management practices associated with antibiotic use, varying globally from farm to farm (24). Hence, details on AMU in dairy animals are more presumptive. According to a survey conducted by the World Organization for Animal Health (OIE) in 2012, only 27% of the OIE member countries adopted an official system for recording AMU in livestock (24).

Antimicrobial usage is the major driving force for developing AMR in animal husbandry. Both commensal and pathogenic bacteria are challenged with antibiotics and in response AMR develops. Drug resistance development has been attributed mainly to suboptimal concentrations of antibiotics in the patients and supplementing subtherapeutic doses of antibiotics to promote growth in food animals (25). It has been observed that bacteria develop resistance through any of the four mechanisms, *viz*., antibiotic inactivation or its modification; alteration in the antibiotic target site; modification in the metabolic pathways to overcome antibiotic effect; and by minimizing entry and/or promoting active efflux of the antibiotic (25). Microbes can build up resistance to antibiotics through mutating existing genes (vertical) (26), or through acquiring new genes from environment, other species, or strains (horizontal gene transfer) (27). The sharing of genetic information between bacteria occurs mainly through mobile genetic elements that includes phages, plasmids, and transposons (28). It has been observed that the resistance among bacterial species is *via* antibiotic-resistant genes and among the major genes leading to AMR includes blaTEM genes for the antibiotics penicillin/amoxicillin/ampicillin (29); *van* for glycopeptides (avoparcin/vancomycin) (30); *erm* gene cluster for macrolides (erythromycin/tylosin/tilmicosin/kitasamycin/oleandomycin) (31); *vatD, vatE, erm* gene cluster, *satA* for streptogramins (virginiamycin/quinupristin-dalfopristin) (31); *sul* genes for sulfonamides (sulfisoxazole/sulfadimethoxine/sulfamethazine) (32); *tet* genes for tetracyclines (chlortetracycline/oxytetracycline/doxycycline) (31); *rgpA–F, mbrA–D* genes for polypeptides (bacitracin); and *cmaA, floR, fexA, fexB, cfr, cat* gene for amphenicols (chloramphenicol) (33).

Presence of resistant pathogenic strains in food matrix creates a direct risk to public health. Food-producing animals are the primary reservoir of zoonotic pathogens. Most frequently encountered resistant pathogenic strains in dairy farming are *Staphylococcus aureus, Escherichia coli, Listeria monocytogenes, Salmonella* spp., etc. *S. aureus* is one among the leading causes of food-borne illnesses. Milk and dairy products are often contaminated with enterotoxigenic strains of *S. aureus*. Therefore, a survey report on the occurrence of *S. aureus* in meat and dairy products indicated around 68.8% strains resistance to at least one antibiotic tested. Usually, *S. aureus* is present on the skin and mucosae of animals, as well as frequently associated with subclinical mastitis, which leads to its entry into milk chain (34). In addition, around 3.75% of these *S. aureus* strains displayed methicillin resistance (35). Sasidharan et al. (36) also found methicillin- and vancomycin-resistant *S. aureus* in dairy products. Jamali and coworkers (37) also tested 2,650 samples of dairy products; out of which *S. aureus* was detected in 12.4% samples in which 16.2% were positive for methicillin resistance.

Besides, *L. monocytogenes* is another resistant bacteria frequently found in dairy products. For instance, oxacillin- and penicillin-resistant *L. monocytogenes* has been reported in dairy products from Lebanon (38). Similarly, a surveillance study carried out in Iran reported MDR *Listeria* spp. in around 7% of traditional dairy products screened in this study (39). Furthermore, antimicrobial-resistant enteric bacteria, mainly *E. coli*, have also been isolated from feces of healthy lactating dairy cattle (40). Shiga toxin-producing MDR *E. coli* strains have also been isolated from cow stool samples in Calcutta, India (41). Similarly, a number of studies have described the occurrence of extended-spectrum β-lactamase producing *E. coli* in food-producing animals. Although, most of these studies are from western countries, quite a number of reports are available from Asia (42, 43). Additionally, antimicrobial-resistant *Salmonella* spp. has also reported in cattle, milk, and milk products. In a study from Ethiopia, around 10.7% of cattle were found positive for MDR *Salmonella* spp. (44).

## Animal–Human Interface

**Figure 2:**
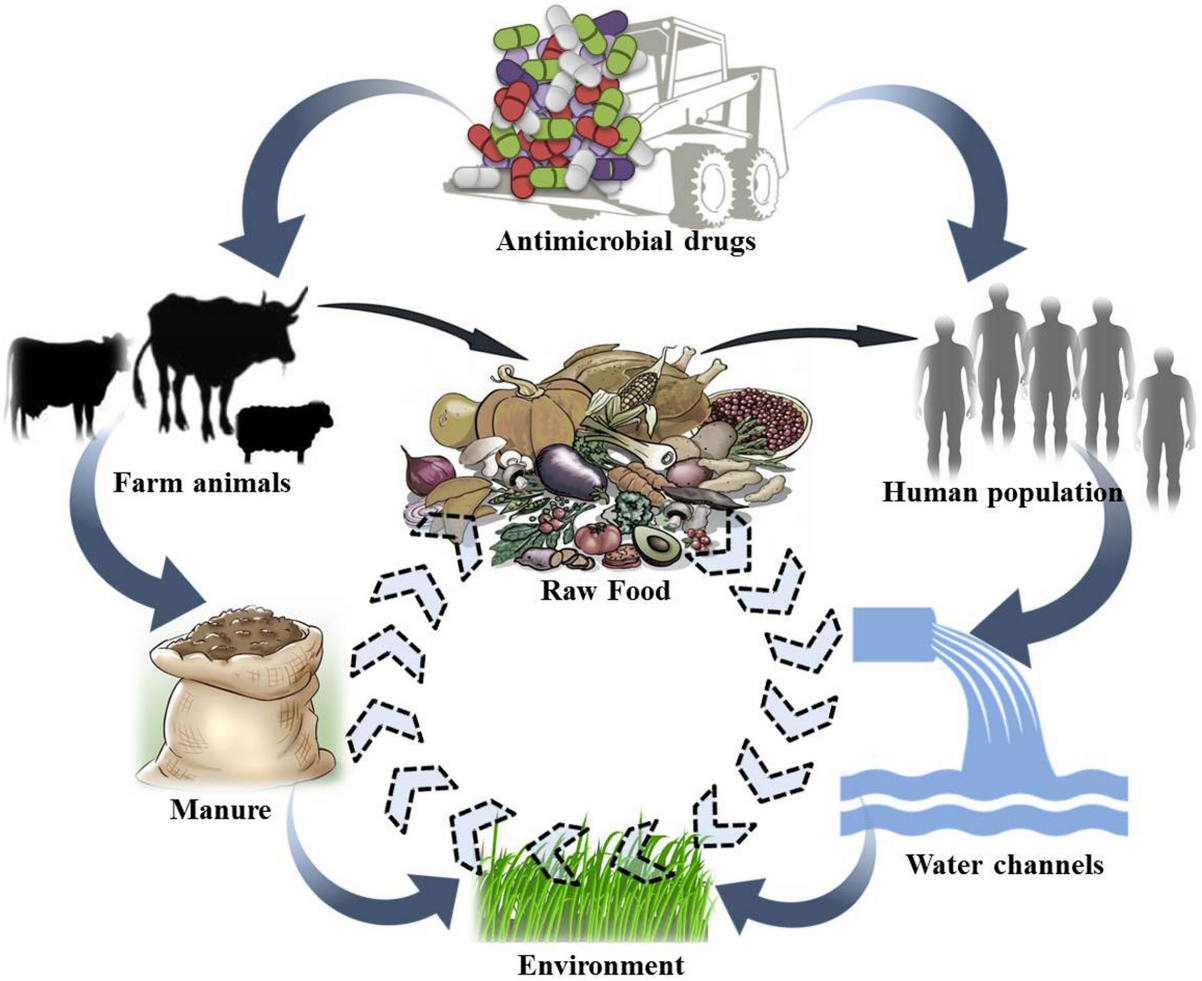
Conceptual representation of possible movement of antibiotic-resistant bacterial strains/genes between different ecosystems.

As observed in human medicine, AMU in veterinary practice, even at a rational dose, may select the genes encoding resistance. These strains now encoding resistance traits can easily transfer to humans, denoting a public health hazard. A reservoir of such strains in dairy animals implies a potential risk for their transfer to humans. Drug-resistant strains of animal origin can spread to humans either through food supply chain (i.e., Meat and Dairy products); direct animal contact; or through environmental routes (18). Several researchers have proposed a relationship between AMU and the occurrence of antimicrobial-resistant strains not only in animals but also in humans having close contact. Any direct or indirect interaction between humans and animals may lead to zoonotic transmission of antibiotic-resistant strains and genes from food animals to humans (Figure 2). Occupationally exposed personnels, *viz*., farmers, food handlers and veterinarians, are more prone to getting colonized or infected with resistant strains (45, 46). Consumers may be exposed to resistant strains and genes through consumption of contaminated food products, i.e., meat, milk, and milk products.

Recently, several reports have portrayed the presence of large number of resistant bacteria and corresponding genes in a variety of animal food products (47, 48). Whole-genome sequencing and phylogenetic studies proposed that the methicillin-resistant *Staphylococcus aureus* (MRSA) in livestock has evolved from methicillin-susceptible *S. aureus* strains of human origin. Quite a few studies have further identified similar or clonally related bacterial strains of animal origin in human populations without any direct exposure to animals, linking them to the consumption and/or handling of food (49). Recently, Horigana et al. (50) studied the risk assessment approach toward the transmission of ESBL-producing *E. coli* from food animals to humans *via* the food chain. Kock and his coworkers (51) also cited that livestock animals frequently transmit *livestock-associated* MRSA to exposed humans. Subsequent cases of infections in humans, resulting from resistant bacteria originating from animal source, are of paramount concern. The problem is more prominent in developing countries, where there are no established guidelines or guidelines that are not followed, and also have high burden of infectious disease along with comparatively low health-care spending (37, 52). MDR bacteria have been isolated from food animals throughout the developing world where AMU is unregulated (20).

## Surveillance and Monitoring

With the mounting pressure of AMR in veterinary system, several countries have initiated surveillance and monitoring programs. In 1997, the World Organization for Animal Health (OIE) proposed standards pertaining to resistance surveillance. The OIE *Terrestrial Animal Health Code* chapter 6.7 talks about the harmonization of surveillance and monitoring programs at national level; chapter 6.8 discusses about monitoring of the AMU patterns in food-producing animals; chapter 6.9 covers the judicious application of antimicrobials in veterinary practice; and chapter 6.10 includes the risk assessment for AMR arising from the AMU in animals (53, 54). These standards were accepted in 2003 along with the introduction of WHO Global Strategy for the containment of AMR. Collaborative consultations between WHO/OIE/FAO experts led to foundation of Codex Alimentarius *Ad Hoc* Inter governmental task force on AMR. This task force proposed the “Guidelines for Risk Analysis of Food-borne Antimicrobial Resistance,” later on adopted by the Codex Alimentarius Commission in 2011 (55).

Surveillance program(s) aims at improved recording of emerging AMR, enhancing the active life of antimicrobial drugs, and providing guidance for the development and usage of newer drugs. Establishment of monitoring program requires consideration of different factors, such as selection of appropriate target bacterial strains to be included, sampling procedures, isolation and susceptibility testing methods, data recording, computing, and reporting. Monitoring and surveillance programs and methodologies followed differ between countries/agencies and are influenced by varied agricultural practices, monitoring needs, and availability of guidelines. Surveillance programs implemented by different countries and agencies have been compiled in Table 1.

**Table 1:** Diverse antimicrobial resistance surveillance and monitoring programs (49, 56, 57).

Country	Regulatory body/Surveillance Program	Link	Testing protocol
Denmark	Danish Integrated Antimicrobial Resistance Monitoring and Research Programme (DANMAP)	http://www.danmap.org	CLSI
United States	National Antimicrobial Resistance Monitoring System (NARMS)	www.cdc.gov/narms/index.html	CLSI
Netherlands	Monitoring of Antimicrobial Resistance and Antibiotic Usage in Animals in the Netherlands (MARAN)	http://www.wageningenur.nl/nl/ExpertisesDienstverlening/Onderzoeksinstituten/Central-Veterinary-Institute/Publicaties-CVI/MARAN-Rapporten.htm	CLSI
Germany	German Resistance Monitoring in Veterinary Medicine (GERM-Vet)	http://vetline.de/17079309/150/3130/69483	CLSI
Canada	Canadian Integrated Program for Antimicrobial Resistance Surveillance (CIPARS)	http://www.phac-aspc.gc.ca/cipars-picra/index-eng.php	CLSI
Italy	Italian Veterinary Antimicrobial Resistance Monitoring programme (ITAVARM)	http://195.45.99.82:800/pdf/itavarm.pdf	CLSI
Japan	Japanese Veterinary Antimicrobial Resistance Monitoring programme (JVARM)	http://www.maff.go.jp/nval/tyosa_kenkyu/taiseiki/monitor/e_index.html	JSC until 2000 CLSI after 2000
Sweden	Swedish Veterinary Antimicrobial Resistance Monitoring programme (SVARM)	http://www.sva.se/en/antibiotika/svarm-reports	SRGA
Spain	Red de Vigilancia de Resistencias Antimicrobialas en Bacterias de Origen Veterinario (VIV)	http://racve.es/publicaciones/red-de-vigilancia-veterinaria-de-resistencias-a-antimicrobianos/	CLSI
Norway	Norwegian Surveillance System for Antimicrobial Drug Resistance (NORM-VET)	https://www.vetinst.no	MIC based automated system
Australia	Pilot surveillance program for antimicrobial resistance in bacteria of animal origin	http://www.health.gov.au/internet/main/publishing.nsf/Content/health-pubhlth-strateg-jetacar-pdf-amrstrategy_affa.htm	CLSI
Finland	The Finnish Veterinary Antimicrobial Resistance Monitoring and Consumption of Antimicrobial Agents report (FINRES-VET)	https://www.evira.fi/globalassets/tietoaevirasta/julkaisut/julkaisusarjat/elaimet/finres_vet_2007_2009.pdf	CLSI
Colombia	Colombian Integrated Program for Antimicrobial Resistance Surveillance (COIPARS)	https://www.ncbi.nlm.nih.gov/pubmed/25903494	CLSI
Mexico	Pilot Integrated Food Chain Surveillance System	https://www.ncbi.nlm.nih.gov/pubmed/18325258 https://www.ncbi.nlm.nih.gov/labs/articles/22870938/	CLSI
28 European countries	Monitoring and analysis of food-borne diseases in Europe (EFSA)	https://www.efsa.europa.eu/en/efsajournal/pub/4380	CLSI
Pan-European (Denmark, Belgium, The Netherlands, The UK, Ireland, Germany, France, Italy, Spain, Poland, Hungary, The Czech Republic)	Centre Europeend’ Etudes pour la Sante Animale (CEESA VetPath)	http://www.ceesa.eu/	CLSI

*JSC, Japanese Society for Chemotherapy; CLSI, Clinical and Laboratory Standards Institute; SRGA, Swedish Reference Group for Antibiotics*.

In order to monitor the status of AMR, the first model of national surveillance program was the *Danish Integrated Antimicrobial Resistance Monitoring and Research Programme* (DANMAP), initiated by the Danish Government in 1995. This program monitors the trend of AMU, status of resistance prevalence and changes over time, and analyzes the link, if any between the usage and resistance development among bacterial strains associated with animals and humans (58). As per the report of DANMAP, 2005 it was observed that from 1999 to 2005 the resistance in *Salmonella* Typhimurium isolated from pigs had increased resistant to tetracycline, sulfonamide, and ampicillin, and these increases were coincided with an increased consumption of tetracycline, sulfonamides, and broad-spectrum penicillin in pigs in the same period. Data from the human population too indicated almost the same pattern, and in the *E. coli* urine isolates resistance to ciprofloxacin, gentamicin, and cefuroxime increased significantly and was consistent with the parallel increases in the consumption of same or similar antimicrobials. These data from DANMAP, 2005 indicate the role of surveillance and monitoring for identification and policy formation at National level.

On similar pattern, *National Antimicrobial Resistance Monitoring System* (NARMS) was constituted in 1996 with joint efforts of the United States Department of Agriculture, FDA, and the Centers for Disease Control and Prevention (CDC). NARMS monitor changes in antimicrobial susceptibilities of zoonotic pathogens from diagnostic specimens (human and animal), healthy farm animals, and from raw product of food-producing animals at slaughter and processing (59). This program deals with studying the prevalence and trends of antimicrobial susceptibility among *Salmonella* spp. and other enteric organisms from the human and animal populations; proper identification of resistance strains; timely delivery of updated information to veterinarians and physicians for increasing the life span of approved antimicrobial drugs; and to properly identify the areas/voids for better research and investigation (60).

In 2009, European Medicines Agency (EMA) launched the *European Surveillance of Veterinary Antimicrobial Consumption* program to monitor AMU in animals from member states. EMA published a report on the sales of veterinary antimicrobial agents, categorized it and reported it in a harmonized manner (61). Apart from the *Centre Européend’ Etudes pour la Santé Animale* (European Animal Health Centre) (CEESA), all the other European veterinary AMR surveillance and monitoring programs are functional at country level. For each of the CEESA programs, isolates are collected from up to nine countries across EU, using uniform collection methodology, followed by four resistance surveillance and monitoring programs, *viz*., VetPath, European Antimicrobial Susceptibility Surveillance in Animals (EASSA), ComPath, and MycoPath. VetPath examines the antimicrobial susceptibility pattern of major disease-causing bacterial pathogens; EASSA covers zoonotic and commensal bacteria; and MycoPath targets disease-causing mycoplasma species in food animals; while ComPath addresses major disease-causing bacterial pathogens in companion animals (56, 62).

The French Agency for Food Safety (*Agence Francaise de Securite Sanitaire des Aliments*, AFSSA) runs two independent surveillance programs which monitors resistance in non-human zoonotic *Salmonella* (AFSSA, Paris), and in bovine pathogenic strains by collecting resistance data from local public veterinary diagnostic laboratories (63).

The Spanish government established *Red de Vigilancia de Resistencias Antimicrobialasen Bacterias de Origen Veterinario* which studies the resistance pattern in microbial strains from sick, healthy, and food animals (64). Additionally, the Veterinary Monitoring of Antimicrobial Resistance (VAV) program has also been established for resistance surveillance and monitoring in Spain (65). In UK, the *Department for Environment, Food and Rural Affairs* compiles AMR data in *Salmonella* spp. The *Canadian Integrated Program for Antimicrobial Resistance Monitoring Surveillance* was designed in light of a 2002 report from the Advisory Committee on Animal Uses of Antimicrobials, and Impact on Resistance and Human Health (66). In Asia, the *Japanese Veterinary Antimicrobial Resistance Monitoring* system was initiated in 1999, and the *Korean Nationwide Surveillance of Antimicrobial Resistance* was established in 1997 in South Korea (67).

In Indian context, there are no regulations for the use of antibiotics in food animals. The Global Antibiotic Resistance Partnership (GARP) was established in the year 2009 to develop actionable policy recommendations for spread of AMR, specifically relevant to low and middle-income countries, including India. In 2011, the India working group report of GARP described the situation of antibiotic usage and emerging resistance and recommended short and long-term actions. The working group recommended establishment of national antibiotic resistance and usage surveillance system, as well as monitoring changes over time. Due to lack of nationwide surveillance mechanism, resistance has gone largely unnoticed in India. However, antibiotic usage trends in India has been increasing steadily, for instance the units of antibiotics sold increased by about 40 per cent between 2005 and 2009 (7). Although increased consumption of antibiotics cannot be directly correlated to developing resistance, their inappropriate use can be. A policy paper of Ministry of Health and Family Welfare, India described means for restraining resistance through minimizing and ensuring rational antibiotic use in animals raised for human consumption; improved surveillance; exploring new drugs; and developing and implementing standard antibiotic policy (68).

Although there is no national database on AMU surveillance in India, few independent studies has been carried out in this regard. In one such study, a very high dependence over flouroquinolones as compared to other antibiotics was reported (69). Moreover, it was observed that the resistance pattern among pathogens differs regionally and data from various studies when combined and evaluated revealed that there is definite resistance to commonly used antibiotics in the pathogens implicated in the disease (*Salmonella* spp., *Shigella* spp., *E. coli, Klebsiella* spp., *Vibrio cholerae, S. aureus, Neisseria* spp., *Klebsiella* spp., *Mycobacterium tuberculosis*, and other strains) (69). It was also reported that Gram-negative bacteria consisting of *Pseudomonas, Acinetobactor, Klebsiella, E. coli*, and *Enterobacter* spp. were resistant to carbapenems, aminoglycosides, fluoroquinolones and third-generation cephalosporins (70). Recently, some newer resistance mechanisms such as the metallo-beta-lactamase NDM-1 have also been witnessed (71, 72). Such resistance mechanisms can further challenge efforts made toward management of AMR.

## Strategies for the Prevention and Containment of AMR

Global co-operative efforts are warranted at individual, community, local, regional, national, and international level to address AMR. In essence, all strategies should target at optimizing the antibiotic usage, minimize un-intended interaction between pathogenic microorganism and antibiotics, limiting the spread of resistant strains, and treating infections with judicious use of antibiotic to affect cure (73). In order to meet this goal, a Tripartite Alliance was formed between the WHO, FAO, and OIE with One Health approach. The tripartite alliance published the Global Action Plan on AMR in 2015. Likewise, FAO also launched its AMR Strategy in 2016 to back the proper execution of the WHO Global Action Plan in food and agricultural sectors (74). The WHO Global Action Plan emphasizes on increased awareness and understanding on AMU and associated AMR; build up knowledge regarding AMR through proper surveillance and research; optimal and rational use of antibiotics; lowering the incidence of infectious diseases; and on organizing resources, research, and development for proper integrated prevention and containment of antibiotic resistance (75).

**Figure 3:**
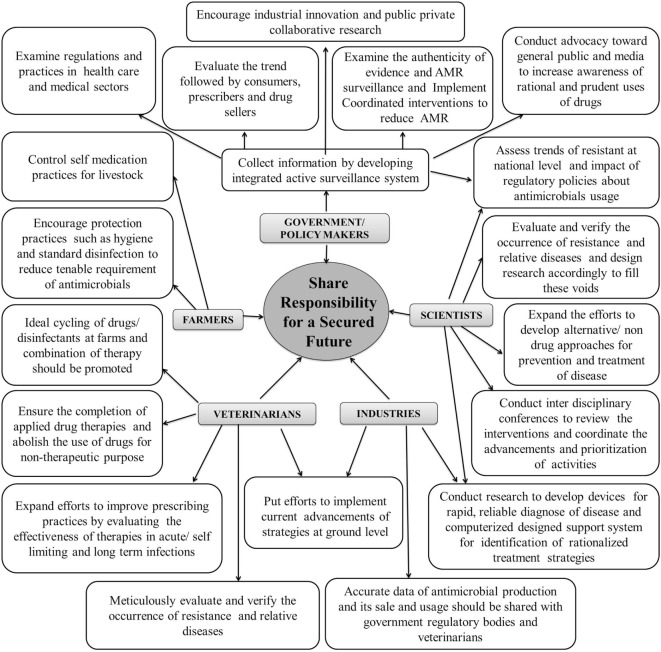
Collaborative meticulous approaches to mitigate antimicrobial resistance (AMR).

Management of AMR in both human and veterinary pathogens requires ideal and concerted action of researchers, policy makers, veterinarian(s), industrialists, and also the end users. Besides development of newer and potent antimicrobials, possible intervention measures that may help in keeping a check at AMR have been compiled in Figure 3. Foremost is the check on AMU through strict legislation and monitoring on over-the-counter without prescription sales. Financial incentives to both the prescriber and dispenser leading to irrational usage needs to be strictly regulated. Periodic updation of standard treatment guidelines into more simple, locally relevant, evidence based, and easy to access documents is essential. Some motivational measures, such as *pay for performance policy* should be implemented by government authorities along with unbiased audit feedback mechanism on drug prescribing rates of individual practitioners and health-care facilities. Infection control interventions need to be re-assessed and improved. National task forces with a far-reaching inter-sectoral coordinating role, involving all relevant stakeholders are desired. Such task forces should outline annual action plans and milestones in different areas such as surveillance, regulation, treatment guidelines, infection control, education, and awareness (76).

## Alternate Management Strategies

**Figure 4:**
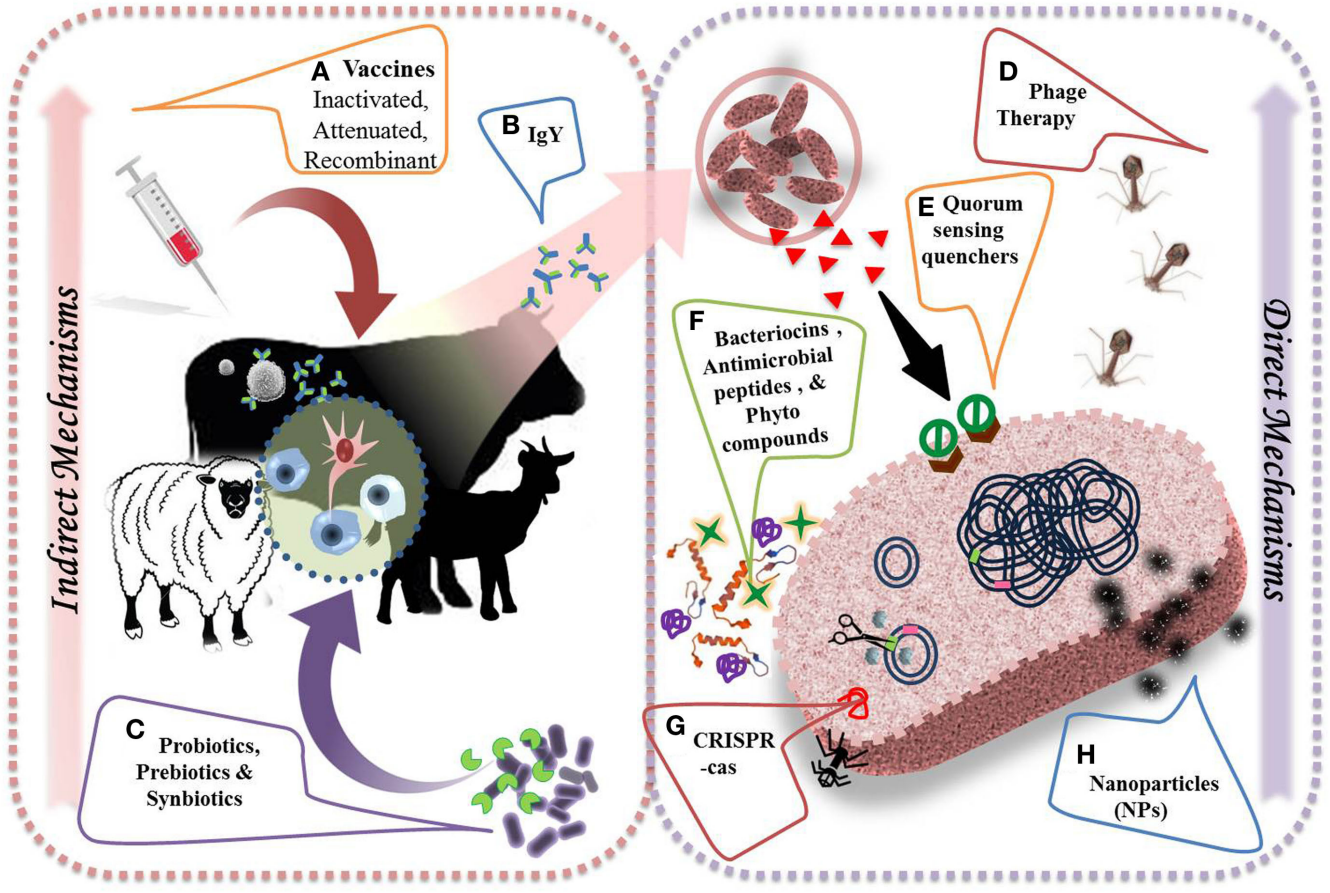
Alternative strategies to combat antimicrobial resistance and their direct and indirect mechanisms of action. **(A)** Vaccination helps in preventing the course of infections by evolving immune cells (i.e. B cells 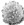, T cells 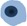) to develop an adaptive immunity by producing specific antibodies (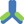) against important pathogens. **(B)** Chicken egg yolk antibodies provide effective treatment approach against several viral and bacterial diseases. **(C)** Probiotics (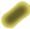), prebiotics (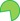), and synbiotics improve general health by selectively stimulating innate immune cells (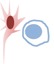). **(D)** Lytic bacteriophage or their purified gene products could be used to treat sepsis and few bacterial infections. **(E)** Quorum sensing quenchers (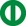) could control virulence of pathogens by inhibiting the binding of auto-inducers (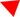) to respective receptors. **(F)** Antimicrobial peptides (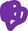), bacteriocins (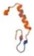), and phytocompounds (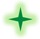) directly inhibit the bacterial growth by acting on bacterial cell membrane. **(G)** Modified CRISPR-Cas approach targets resistance genes in pathogens and reverse the selective pressure of resistance. **(H)** Metal-based nanoparticles (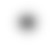) help in blockage of enzyme pathways, alteration of cell wall, and nucleic material pathways.

Spread of AMR in conjunction with slow emergence of novel antibiotics; have created a deficit of effective therapies against microbial disorders. Presently, the mobility of a newer drug from the phase of discovery, clinical efficacy, and safety assessment to approval is estimated to cost more than double (77, 78). Therefore, finding and introducing novel antibiotics has become more challenging and antimicrobial research no longer remains an attractive option for investors seeking quick and substantial returns. It is high time for more innovative and bold solutions to curtail resistance to antibiotics and speed-up the discovery and introduction of new, safe, resistance-free and economical alternatives to antibiotics (49). Ideal alternatives to antibiotics should be non-toxic with easy elimination from the body, stable through gastrointestinal transit, easily decomposed and environment friendly, selectively active against pathogens with minimum or no effect over resident gut flora, improve feed efficiency and promote animal growth, and above all free from resistance (79). Novel strategies, showing promise toward replacing and/or serving as an adjunct to current day antibiotics and displaying activity against MDR strains have been discussed in detail below in this section (Figure 4).

## Vaccines

**Figure 5:**
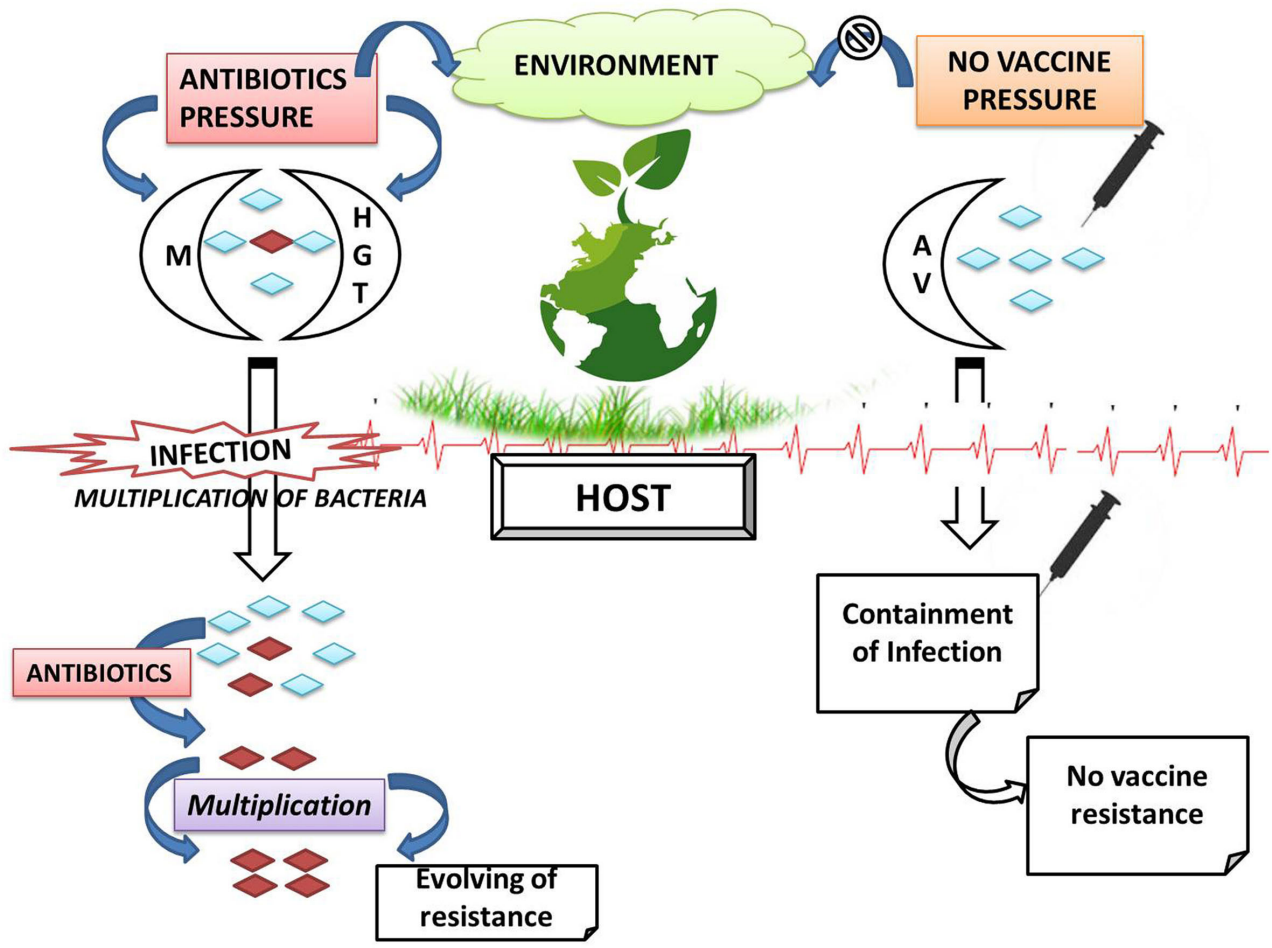
Comparative resistant phenomenon with respect to antibiotics and vaccines (M, mutation; HGT, horizontal gene transfer; AV, antigenic variation; 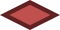, antibiotic-resistant bacteria; 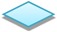 antibiotic-sensitive bacteria).

Vaccination is used as a powerful strategy for prevention and even eradication of infectious diseases. Vaccination is promising in eradicating many diseases worldwide, *viz*., small pox and polio, successfully and, thus, could be used to restrain AMR bacteria especially having wide impact on human and animal health. The application of vaccines as a valid alternative therapeutic to antibiotics has attracted much attention due to the fact that resistance is not observed against vaccines, because of its characteristic features and mechanism of action (80). Recombinant vaccines generally possess multiple immunogenic epitopes, thereby requiring multiple mutations for allowing resistance development and single mutation vaccines are not acceptable clinically. Vaccines developed though recombinant methods restrict bacterial replication, prevent selection of variants, and overcome selection pressure in environment (Figure 5). Vaccines are biotic preparations that develop acquired immunity against a selective organism/target (Figure 4) (81–83). In general, vaccines falls under three categories, *viz*., killed/*inactivated vaccines* (antigens with adjuvants); live/*attenuated vaccines* (live vaccines), and *recombinant vaccines* (subunit antigens or genetically engineered organisms). In veterinary practice, multivalent/multicomponent vaccine approach has been mostly explored. Veterinary vaccines are ideal candidate for animal health and welfare and for food production as they help in preventing the infection; reducing consumption of antimicrobial drugs; enhancing food productivity; and mitigating the impacts of antibiotic resistance. Vaccination too lessens the transmission of zoonotic and foodborne infections to humans (49, 84).

Keeping in mind the utility of vaccines over antibiotics, R&D is being undertaken to produce advanced vaccines through recombinant DNA technology. Recombinant veterinary vaccines are classified into three groups, i.e., DNA or RNA vaccines, subunit recombinant vaccines, and vectored vaccines. Recombinant vaccine development involves cloning of DNA fragment into a vector. Recombinant DNA or RNA vaccines results in expression of pathogen specific proteins, while, in subunit vaccines a recombinant protein is generated in laboratory and injected into the host. Recombinant vector vaccines use an attenuated bacterium or virus to either multiply and express the antigen within the host or introduce DNA in the host cells (85). Further advancement in genetic engineering and bioinformatics permits the identification and synthesis of epitopes possessing ability to trigger the immune response, resulting in the development of recombinant vaccines. Such recombinant vaccines provide targeted immunity and eliminate the use of attenuated infectious agents, ensure safety, and early onset of immunity (85). These vaccines comprise of minimum of one modified antigen having ability to induce an immune response against targeted pathogens upon co-administration with adjuvants or plasmids (86).

The first DNA vaccine for veterinary application was licensed in 2005, against the Equine West Nile Virus (87, 88). Subunit vaccines, namely Gavac and Tick GUARD, developed by Heber Biotec and Fort Dodge too have been approved for commercial use in cattle against *Ripcephalus (Boophilus) microplus*. In India, Shakya et al. (89) characterized recombinant Subolesin, having 44 and 37.2% vaccine efficacy against female ticks in crossbred cattle male calves after first and second challenges, respectively. The subolesin ortholog of *Hyalomma anatolicum* and *R*. (B) *microplus* was reported to be highly conserved across different Indian strains, having over 50% of sequence homology at the amino acid level. HIDATIL EG95, a subunit vaccine, is available for protecting sheep and goats against *Echinococcus granulosus* (85). Leish-Tec is one of the commercially available recombinant vaccines against protozoans (90). Among vectored vaccines, cattle vaccinated with a BCG strain overexpressing the Ag85B antigen (protein found on the bacterial surface) displayed enhanced protective immune responses against *Mycobacterium bovis* (91). Currently, most of the practiced bacterial vaccines include live attenuated and inactivated or killed microbial strains, with varying degrees of efficacy, e.g., RB-51, a commercially available bacterial vaccines used to cure *Brucella abortus* in cattles (49). Regarding commercialization of veterinary vaccines in India, till date no recombinant vaccine has been found to satisfy regulating agencies and so has not been in the Indian market.

In the present scenario, research is inclined toward exploring prophylactic vaccines as therapeutics. Bacterin, a suspension of killed or attenuated bacteria is active against multiple pathogens (92). Globally, bovine mastitis is a major concern for dairy farming. *S. aureus* is one of the organisms associated with mastitis. A number of approaches, such as whole organism vaccines, live attenuated *S. aureus*, capsular polysaccharide–protein conjugate vaccines, DNA vaccines encoding clumping factor A, and recombinant *S. aureus*-mutated enterotoxin type C have been explored for vaccine development against *S. aureus* (93). Earlier, Sayed et al. (94) studied the anti-mastitis efficacy of a locally prepared polyvalent vaccine (Montanide ISA-206 adjuvanted inactivated polyvalent vaccine) comprising of *S. aureus, Streptococcus agalactiae*, and *E. coli*. The study reported an improved immune response in the vaccinated group. Additionally, bacterial strains, such as *Salmonella* Typhimurium and lactic acid bacteria (LAB), can be explored as a vehicle for vaccine delivery due to specific targeting and activation of APCs (antigen-presenting cells) (48, 88, 95). Cloven-hoofed animals including dairy animals, *viz*., cattle, buffalo, sheep, and goat, are susceptible to foot-and-mouth disease (FMD), caused by a fast-replicating FMD virus. Inactivated whole virus vaccines are currently being explored for preventing FMD; however, faces several short-falls including risk of escape of live virus and requirement of costly biocontainment facilities. Virus-like particles, being structurally similar to virus, but being non-infectious, safe, and having good immunogenic properties are being explored as an alternate to whole virus vaccines. Recently Kumar et al. (96) constructed and characterized recombinant adenovirus expressing capsid proteins of Indian FMD virus strain O/IND/R2/75 to a high titer. Later, Eri silkworm larvae were explored for production of VLPs of FMD O/IND/R2/75 using recombinant baculovirus encoding polyprotein of FMD virus (97).

Although vaccination has emerged as a powerful tool against drug resistance strains, several evolving strains may escape immunity induced by vaccine administration. Hence, a regular update on vaccine strain is warranted (98). Ideally, it could be possible to develop and use vaccines against an etiological agent having reportedly high endemicity, having stable genetic composition and where chances of AMR are very high. Though this strategy will involve huge expenditure as development of vaccine against a homologous strain could be expensive, it could provide for an alternative we look for. Advanced recombinant vaccines are undoubtedly the future of addressing infectious disease in dairy animals. There is a need to develop safer, potent, and better characterized vaccines with broader protection against multiple pathogens. However, developed vaccines needs to qualify rules and registration guidelines for recombinant vaccines. The biological characteristics of recombinant virus must be documented for proper risk assessment associated with its use in target species. Safety characteristics of such vaccines must be ascertained for both host and environment before field trails can be authorized (99).

## Phytocompounds

Since time immemorial, plants have been indispensable sources of traditional medicine for the management of human and livestock health. Livestock farmers, particularly from rural areas consult or visits traditional practioners for the treatment of various animals disorders in developing countries (100). Traditional remedies, mainly comprising of plant components/extracts, being natural, non-toxic, inexpensive, and easily available are readily accepted by the farming community. Ethnoveterinary medicine refers to the holistic and interdisciplinary study of traditional knowledge, practices, and methods pertaining to animal health care (101). Plants possess multifunctional properties, primarily linked to the presence of varied bioactive secondary metabolites or phytocompounds, *viz*., glycosides and alkaloids (alcohols, aldehydes, esters, ethers, ketones, lactones, etc.), anthocyanins, coumarins, flavonoids, phenolics (tannins), saponins, and terpenoids (mono and sesquiterpenes, steroids, etc.) (102). Several plant components are incorporated to animal feed as growth promoters and health protectants (79, 103, 104). Hence, any potent phytocompound displaying broad-spectrum antimicrobial activity can serve as an ideal alternative to conventional veterinary antibiotics (105, 106).

Plant extracts or phytobiotics are mainly explored in animal nutrition for their diverse pharmacological activities, such as antimicrobial, anti-inflammatory, antioxidative properties, etc. Plant extracts, such as tea tree oil and its active component, terpinen-4-ol, have demonstrated efficacy in bovine mastitis treatment. Terpinen-4-ol inhibits pro-inflammatory cytokine, upregulates anti-inflammatory cytokine expression, and displays tissue healing characteristics (107). Ghosh et al. (108) documented the antioxidant, anti-inflammatory, hypoglycemic, anticancer, and antimicrobial properties of diferuloylmethane, a polyphenol isolated from turmeric (*Curcuma longa*) rhizomes. Brooks et al. (109) reported enhanced expression of CD4^+^ and CD8^+^ T cells in neonatal calves fed with extracts from *Morinda citrifolia*. The plant extracts also possess bactericidal and immunomodulatory properties, and positively impacts animal growth performance (110). Extracts from *Allium sativum* also exhibits antibacterial, anti-diarrheal, anti-inflammatory, and immune-modulatory properties (Figure 4). In a trial involving neonatal calves, allicin delayed the onset of *Cryptosporidium parvum*-induced diarrhea. Similar findings were recorded in pre-ruminant calves (111, 112).

Earlier, Luseba and Tshisikhawe (113) reviewed the available data on medicinal plants used for the treatment of cattle disorders in South Africa. Whole plant, leaves, and root extracts from *Asparagus falcatus, Tagetes minuta, Diospyro slycioides*, and *Vernonia corymbosa* have been shown to control ticks and worms in cattle. *Elephantor rhizaburkei* bulb and *Xanthocersis zambesiaca* bark manages diarrheal problems in cattle. In another study, Panda and Dhal (114) reviewed the veterinary medicinal plants of Odisha, India. Various plants and their components have proposed role in management of cattle diseases, including diarrhea, lactation, and foot-and-mouth disorders. On similar pattern, Narayana and Rao (101) reviewed the ethnomedicinal plants of Andhra Pradesh, India. They documented several preventive and therapeutic roles of stem bark, leaves, rhizome, and whole plant extracts of several plants for curing anthrax, dysentery, wounds, ephemeral fever, FMDs, bronchial disorders, gout/inflammation, fractures, etc. Earlier, Dhama and coworkers (102) reviewed and compiled the available literature on antibacterial activity of medicinal plants and herbs. Few of the follow up studies have been recorded in Table 2.

**Table 2:** Plant extracts explored against various diseases causing bacterial pathogens in dairy animals.

Scientific name (common name)	Plant part used	Type of extracts	Pharmacologically active phytoconstituents	Antibacterial activity against	Reference
*Senna macranthera*	Roots	Dichloromethane	Emodine, physione, and chrysophanol	*Staphylococcus aureus*	(115)
*Combretum molle* (velvet leaved Combretum), *Xanthium strumarium* (Cockleba)	Stem, bark, leaves	95% ethanol	–	*S. aureus* and *Streptococcus agalactiae*	(116)
*Allium sativum* (garlic)	Cloves	Juice	–	*Cryptosporidium* sp.	(117)
*Neoglaziovia variegata*	Leaves	Hexane and ethanolic	–	*Rhipicephalus (Boophilus) microplus*	(118)
*Psidium guajava* (guava) *T. foeum-graecum* (fenugreek)	Leaves, seeds	Methanol	–	*S. aureus, Escherichia coli, Pseudomonas aeruginosa, Salmonella* sp.	(119)
*Thalictrum minus*	Roots	Dichloromethane and methanol (1:1)	Benzylisoquinoline alkaloids (5’- hydroxythalidasine, thalrugosaminine, *O*-methylthalicberine)	*Staphylococcus xylosus, S. lentus, S. equorum, Enterococcus faecalis, E. coli*	(120)
*Cinnamon cassia* oil	–	–	–	*S. aureus, S. epidermidis, S. hyicus, S. xylosus, E. coli*	(121)
*Holarrhena antidysenterica*	Bark	Ethanolic	–	*E. coli*	(122)
*A. sativum, Bunium persicum, Oryza sativa, Triticum aestivum*	Bulb, seeds, fruits	Methanolic	Alkaloids	*S. aureus, E. coli, K. pneumoniae*	(123)
*Dalbergia retusa, Crescential alata, P. guajava, Vitex mollis*	Leaves	Methanolic	–	Methicillin-resistant *S. aureus*	(124)
*Acacia nilotica, Tetradenia riparia*	Bark, flower	Acetone	–	*S. aureus, Streptococcus uberis, S. agalactiae, K. pneumoniae, E. coli, P. aeruginosa, P. mirabilis*	(125)
*Salvadora persica, Colophospermum mopane, Dichrostachys cinerea*	Leaves, bark, roots	Methanolic	–	*S. aureus, E. coli*	(126)
*Aloe vera, Curcuma longa*	–	Aqueous, ethanol, and ethyl acetate	–	*E. coli, S. aureus, P. aeruginosa*	(127)
*Evernia prunastri* (plum lichen), *Artemisia absinthium* (Absinthe wormwood), *Lavandula angustifolia* (Lavender)	–	96% Ethanol	–	*S. aureus, S. xylosus, S. intermedius, S. chromogenes, S. hyicus, Vibrio fluvialis, Serratia liquefaciens, E. coli, Lactococcus lactis, Enterobacter intermedius, Bacillus cereus, Yersinia ruckeri, Aeromonas hydrophila, Kytococcus sedentarius*	(128)
*Panicum turgidum* (Thummam)	–	Aqueous	–	*Streptococcus pyogens, Candida albicans*	(129)

Besides other plant components, use of plant essential oils (EOs) for the management of livestock is becoming a common practice. Nanon et al. (130) documented that EOs from lemon grass and mixture of garlic and ginger can be explored as rumen modifier, resulting in improved feed digestion, particularly of roughage feeds. A recent study conducted by Jeshari et al. (131) showed that supplementing starter diets with a mixture of EOs from *Rosmarinus officinalis* L., *Zataria multiflora* Boiss, and *Mentha pulegium* L. positively impacted the growth performance of suckling calves. Plant-food by-products are also being utilized as animal feed (132, 133). Pomegranate byproduct extracts act as a good source of phenolics, having inherent antioxidant, anti-inflammatory, and antimicrobial activity (134). In past, several phyto-compunds have displayed promising antibacterial activity against MDR strains. To name a few, extacts from 18 herbal plants considerably limited the growth of MDR *Acinetobacter baumannii* under *in vitro* conditions (135). Recently, phytocompounds have been reported to display potent antibacterial activity against MRSA (136) and extended-spectrum beta-lactamase-producing bacteria (137, 138). Interest toward adopting plant-based compounds as therapeutics is gaining interest. However, most of the results are coming from *in vitro* studies and required further *in vivo* studies for validation.

The mechanisms underlying the antibacterial properties of phytocompounds along with their impacts on pathogen virulence, and other host factors have been reviewed in detail earlier by Dhama et al. (102). In brief, the antimicrobial activity, whether bactericidal or bacteriostatic, may be mediated through enhanced immunity, proliferation of B and T cells, stress amelioration, cytokine regulation, suppressing free radicals, inhibition of prostaglandin biosynthesis, decreased production of inflammatory molecules (histamine, serotonin), enhanced cortisol activity, improved blood circulation promoting toxin removal from body, NF-κB downregulation, damage to cell membrane with loss of cytoplasmic contents and electrolytes, quorum sensing (QS) inhibition with minimized synthesis of LasA protease, LasB elastase and N-acyl homoserine lactone (AHL) molecules, and promoting phagocytosis, etc. (102).

The main constraints associated with application of phytocompounds in animal husbandry, particularly dairy animals is its being a complex blend of bioactive compounds and variation in composition due to several biological, processing, and storage factors (139). Further, the units of application are not standardized. Future research targeting phytocompounds purification, understanding their mode of action, standardizing appropriate units of administration, compatibility with diet, toxicity, safety and stability assessment, as well as their pharmacodynamics and pharmacokinetic properties is required for establishing them as an effective alternate to antibiotics.

## Probiotics, Prebiotics, and Synbiotics

The ban of dietary antimicrobial agents attracted a great deal of attention toward the application of probiotics, prebiotics, and synbiotics for production of safe food along with improved gut health (140). With the advent of new antibiotic resistance strains, probiotic strains are gaining popularity in both medical and livestock sector (141–143). In few cases, probiotics and direct-fed microbials (DFMs) have been employed as animal feed that acts as an alternative to antibiotics, as growth promoters, and also control enteric pathogens (144). Several reports propose substitution of antibiotics with probiotic strains as growth promoters in livestock animals (144, 145). Probiotics as a microbial food supplement helps in maintaining the intestinal microbial balance of host and also act as immune-stimulant (Figure 4) (146, 147). Probiotic additives have been explored to improve animal health and productivity by stimulating healthy intestinal microbial ecosystem (148, 149), promoting digestion, nutrient absorption, and bioavailability (150), preventing enteric pathogens colonization (151), restoring gut microflora (152), lowering pH, and improving mucosal immunity (149). Therefore, probiotics can act as an ideal candidate to improve the general health and productivity of ruminants (153).

It is quite evident from available data that probiotics have direct antagonistic activity against varied number of resistant strains. Jamalifar et al. (154) reported strong antagonistic activity of *Lactobacillus acidophilus* fecal isolate against the MDR clinical isolate of *Pseudomonas aeruginosa*. On similar lines, *Bifidobacterium pseudocatenulatum* SPM1309 was shown to inhibit MDR *P. aeruginosa* and *A. baumannii* (155). *Lactobacillus casei* strain exhibited strong activities against MDR *Shigella* strain (156). Strains of *L. casei* also displayed strong inhibitory effect against MDR *E. coli* (157). In a recent study, Kumar et al. (97) documented antimicrobial activity of *Lactobacillus plantarum* and *L. acidophilus* against MDR entero-aggregative *E. coli*. Ripamonti and coworkers reported inhibitory effect of a species-specific probiotic formulation against MDR *E. coli* isolates from calves (158). *Lactobacillus* sp., *Bacillus* sp., *Enterococcus* sp., *Bifidobacterium* sp., *Lactococcus* sp., *Pediococcus* sp., *Streptococcus* sp., *Propionibacterium* sp., *Saccharomyces* sp., and *Aspergillus* sp. are mainly used as candidate probiotics in livestock and animal husbandry, either as feed additive or in some other form (79, 153, 159, 160).

Gulbe et al. (161) documented the synergistic antagonistic effect of *Lactobacillus helveticus* and lysozyme against mastitis-causing staphylococci. Probiotic strains have also been reported to mitigate *E. coli* O157:H7 in cattle. A probiotic mix of *Streptococcus bovis* and *L. gallinarum* resulted in reduced *E. coli* O157 shedding in experimentally infected calves (162). A blend of *L. acidilactici* and *Pediococcus* sp. of bovine origin was shown to directly inhibit *E. coli* O157:H7 under *in vitro* conditions (163). *Lactobacillus* sp. based DFMs, currently marketed as Bovamine™ and BovamineDefend™ are being widely explored for reducing *E. coli* O157:H7 in cattle (144).

Prebiotics are the non-digestible food ingredients that are selectively metabolized by the gut microbial inhabitants. They support selectively proliferation of intestinal bacteria, promote immune response, display antimicrobial activities, and deliver beneficial health effect to host. The most commonly used prebiotics are carbohydrate substrates, i.e., oligosaccharides, polysaccharides, polyols, protein hydrolyzates, etc. (79, 149). Prebiotics have long history of use as feed additives. Some oligosaccharides have been proposed to have specific health benefits in calves. To name a few, mannan-oligosaccharide, a complex mannose sugar is believed to block pathogen colonization in the intestinal tract. It has been observed that few prebiotics endow competitive advantage to selective beneficial gut flora, i.e., probiotic strains such as, *Bifidobacterium* sp., *Lactobacillus* sp., that are known to act antagonistically against pathogens (164, 165). Fructooligosaccharide along with spray-dried bovine serum minimized the incidence and severity of enteric disease in calves (166). This attribute has been linked to the pathogen exclusion properties of fructooligosaccharide, wherein the *E. coli* and *Salmonella* adhesion to the intestinal epithelium is restricted (167). The administration of oligosaccharides to weaned calves promotes desirable intestinal (rumen and/or lower intestine) community that prepares ground for improved growth performance at an older age. Celmanax, a commercial prebiotic for cattle prevent enterohemorrhagic *E*. *coli* colonization in the intestine (168).

A combinantion of probiotics and prebiotics, recognized as synbiotics is designed to avail health benefits in a highly targeted fashion (169). It has been found that synbiotic supplementation to animal feed augments the lactate and antibody production, lowers intestinal pH, which alters the intestinal microflora, and minimizes harmful bacteria in the gut (170). Earlier, Bomba et al. (171) also reported synergistic role of synbiotics in reducing pathogenic bacteria in food animals. In an *in vivo* study, continuous oral co-administration of *Bifidobacterium breve* strain Yakult and galacto-oligosaccharide rendered protection against MDR *A. baumannii* and increased the survival rates of mice. Synbiotic treatment promoted the indigenous gut flora from pathogenic one, reduced endotoxin level and pathogen mediated damage, and rejuvenated the gut barrier function. No positive effects were recorded upon treatment with GOS alone (172). Continuous antibiotic treatment usually results in washing out of indigenous gut flora, which can be replenished with the concomitant administration of selective probiotic and prebiotic components.

Although the prospects of using pro-, pre-, and syn- biotics in dairy animals are bright, their current application is limited by paucity of studies answering the strain-specific activity of probiotic strains; variability within host animal species; age, diet, and physical condition of target animal; degree of the pathogen challenge and other stressors; dose and duration of administration, etc. All these factors need to be considered and standardized. Also there is a need to meticulously understand the safety characteristic of probiotic strains, especially with regard to possibility of transfer of resistance traits, and in immune-compromised subjects. Few recent studies have documented the resistance profile of *Lactobacillus* spp. strains from different origin (173). Naturally being safe and supported with current research findings, further involvement of omics tools will strengthen their candidature as a possible application in food animals, as a preventive and supportive therapy, as an adjunct to antibiotics or even as a safe alternate to current day antibiotics, reducing the AMU and associated resistance.

## Bacteriocins

Bacteriocins are the ribosomally synthesized bacterial antimicrobial peptides (AMPs) that can kill or inhibit closely related bacterial strains (174). They are generally produced by a variety of Gram-positive bacteria and classified into two classes, i.e., *class I*, also called lantibiotics because they are heavily modified after translation to contain amino acids, such as lanthionine and B-methyllanthionine, and *class II*, heat-stable peptides, which are released without any posttranslational modifications (175). Most commonly, LAB are known to secrete bacteriocins, i.e., nisin (*Lactococcus lactis*), lactocin (*Lactobacillus sakei*), pediocin (*Pediococcus acidilactici*), acidocin (*L. acidophilus*), sacacin (*L. sakei*), plantaricin (*L. plantarum*), helveticin (*L. helveticus*), curvacin (*L. curvatus*), lactobin (*L. amylovorus*), etc. Bacteriocins produced by Gram-negative bacteria are colicins and microcins (*E. coli*). Among LAB bacteriocins, nisin is the most commonly explored (176, 177). Diverse classes of bacteriocins have varied mechanisms of action which are different from antibiotics (178). Applications of bacteriocins are widespread; ranging from treatment of topical and internal infections, food preservation and antimicrobial packaging.

The mechanism of action divides bacteriocins in to two distinct types; one those work on cell envelope and other those inhibit gene and protein expression (Figure 4). Several investigators have demonstrated that application of these bacteriocins with combination of antibiotics significantly improves the effectiveness of drugs. For instance, Tong et al. (179) have examined the minimum inhibitory concentration of 18 different antibiotics against *Enterococcus faecalis* and found that addition of nisin reduced the MIC values of all the tested drugs. In addition biofilm forming pathogens such as *S. aureus*, associated with bovine mastitis has also been mitigated with the combination of nisin and lysostaphin (180). Interestingly, McCaughey et al. (181) have reported that a *P. aeruginosa*-specific bacteriocin, pyocins S5, was effective at 100-fold lower concentration than the most commonly used inhaled antibiotic tobramycin in a murine model.

Furthermore, bacteriocins based products are commercially available for the treatment of superficial and systemic bacterial infections and have several potential applications in the veterinary field also. For example, nisin based teat sanitizers, Amibicin N^®^ (Applied Microbiology, Inc., New York, NY, USA), Wipe-Out^®^ Dairy Wipes, and Mast Out^®^ (Immucell Corporation) are already in practice and serving as an effective alternative for the treatment of mastitis (182). Similarly, a teat dip containing lantibiotic, lacticin 3147, is also available for therapeutic remediation of staphylococcal infection. This product has been proven significantly effective against *S. aureus, S. dysgalactiae*, and *Streptococcus uberis* (183). Likewise, two broad-spectrum peptides/bacteriocins, geobacillus I and geobacillus II, were characterized by Rea et al. (184) and were found to be very effective against *S. dysgalactiae* subsp. *dysgalactiae*. There are few other potential examples, such as nisin U, nisin Z, uberolysin, bacteriocin ST91KM, morricin 269, kurstacin 287, kenyacin 404, entomocin, Pep5, epidermin, epilancin K7, epicidin 280 and aureocins A70, A53, and 215FN, which have displayed potential activity against *S. aureus* and *S. agalactiae* (182, 185–189). Beside skin and udder infections, bacteriocins are also proven to be effective against a range of enteropathogens. Microcin C7 and Colicins 1b and E1 from *E. coli* (190) as well as enterocin RM6 inhibits pathogenic enterobacteria, i.e., *Enterobacter agglomerans, E. coli, K. pneumoniae, Morganella morganii, Salmonella enterica, Shigella flexneri* and *Yersinia enterocolitica* (191).

Therefore, bacteriocins can be explored for controlling bacterial infections in livestock animals, including mastitis and other systemic infection, which could reduce the dependency on antibiotics for the treatment. However, the major concern regarding bacteriocin is their narrow spectrum efficacy along with insufficient toxicity data. Therefore, the future role of bacteriocins as an alternative therapy to classical antibiotics is still in its infancy. Moreover, the higher specificity of action of bacteriocins, especially against MDR strains is very encouraging. In future, the introduction of chemical and genetic engeneering in order to auspicious consideration of bacteriocins as a consistent treatment strategy will offer a strong solution for growing AMR problem.

## Antimicrobial Peptides

Antimicrobial Peptides, recognized as host defense peptides, are one among the leading alternatives to ongoing antibiotic therapy and are abundantly distributed in nature (192). They are ubiquitously present in all the organisms and possess varied structural and functional diversity and displays remarkable antimicrobial and immunomodulatory properties, which make them an ideal candidate for the development of novel therapeutics. AMPs may be either anionic (a small group present in ruminants, mainly rich in aspartate and glutamate) or cationic (a large group present in all domesticated animals). Cationic peptides may be linear, helical, proline-rich linear peptides, and cysteine-stabilized peptides with a β-sheet. Usually they exhibit broad-spectrum antimicrobial activity against bacteria and fungi (193). Further, based on the peptide synthesis machinery, AMPs may be either ribosomally (bacteriocins) or non-ribosomally (bacitracin, gramicidin, polymyxin, etc.) synthesized (79). These small polypeptides are amphipathic in nature and exhibit hydrophobic/cationic properties which promote their intercalation into the bacterial phospholipid bilayer, creating pores and resulting in osmotic lysis (Figure 4) (193). In addition, they have the capability to bypass the common resistance mechanisms employed against standard antibiotics. Besides carrying potent antimicrobial activities, AMPs promotes the nutrient digestibility, intestinal morphology, and beneficial gut microbiota, having overall positive effects on growth performance of animals (83). In one such study, AMPs, A3 and P5 (synthetic) and cecropin AD (artificial), positively regulated the growth performance of animals mediated through selection of beneficial gut microbiota (194). Ruminants possess vast array of AMPs that offers a natural innate barrier against microbial pathogens (195). In contrast to antibiotics, AMPs usually targets the host cells and prevents infection in an indirect mode, conferring an additional advantage over antibiotics (83).

It has been observed that AMP Esc-1a (1-21) NH_2_ has potent and rapid activity against bovine mastitis pathogen, *S. agalactiae* (196). Bovine AMPs (Peptide B/enkelytin) are well studied and exhibit potent antimicrobial efficacy. Processing of these peptides in brain plays an important role in neuro-immune responses to bacterial invasion; thereby acting as an important class of immune-modulatory peptides (197). Bovine AMP, Kappacin, was initially isolated from milk and cheese and is the cleavage product of caseinomacropeptide with no posttranslational modifications. It displays broad range of activity in a pH-dependent manner (198, 199). Strub et al. (200) studied another bovine AMP, Chromacin, isolated from bovine chromaffin granules, which inhibits the growth of both Gram-positive and Gram-negative bacteria. Bacitracin methylene salicylic acid and bacitracin zinc had been approved in USA and China as feed additives (79). In an interesting study, Zhang et al. (201) observed that AMPs can be explored to overcome mastitis by applying recombinant DNA technology, which enables mammary cells to synthesize and secrete lactoferricin and tracheal antibacterial peptides, having role in management of mastitis. AMPs have also been screened for their antimicrobial potency against pathogens exhibiting multidrug resistance and had been proposed as an alternative to antibiotics in animal husbandry (79). Reports emerging from research studies applying AMPs in ruminants are reviewed elsewhere (193).

Apart from AMPs, milk proteins acts as precursors for many biologically active peptides, including antimicrobial ones. These milk-derived peptides have already been considered for application both as dietary supplements in functional foods and as drugs (202). The number of identified and characterized milk-based bioactive peptides is slowly increasing. Although a number of milk-derived AMPs are now well characterized, key information regarding their structural and functional attributes is still unavailable to potential users. Theolier and coworkers (203) established a milk AMP database that contains valuable information including microbiological and physicochemical data on AMPs of milk protein origin. The database comprised of 371 entries in 2014, including hydrolyzates (9), AMPs (299), peptides predicted as antimicrobial (23), and non-active peptides (40) (203); and has emerged to 944 entries in 2017, as recently reviewed by Nielsen et al. (204). This information would facilitate the study of the potential of these peptides to thwart antibiotic resistance in pathogenic bacteria. Mandal et al. (205) observed the antimicrobial, antioxidant, and growth-stimulating activity of 24 fractions of peptides purified from human milk. Among them, two peptides (f8 and f12) identified as lactoferrin-derived peptide and kappa casein short-chain peptide displayed broad-spectrum antimicrobial activities. In a different study, mass spectrometry based analysis of the human milk peptidome identified almost 700 endogenous peptides from 30 different proteins, resulting from remarkably specific and well-conserved endogenous proteolytic activity of mammary glands (206).

With the discovery of naturally occurring and synthetic AMPs, attention is being extended to assess antibacterial activity of their structural modifications. Amino acid substitution is one among the simplest approach for enhancing biological activity, as well as reducing cytotoxicity of AMPs (207). In one such study, Lee and coworkers (207), designed a helix-PXXP-helix (HPA3P2) structure, wherein glutamine and phenylalanine replaced proline of HPA3 at 9th and 12th position, respectively. The structural modification dramatically increased the antibacterial activity under *in vivo* conditions. The modified peptide was also tested for antimicrobial efficacy against MDR *P. aeruginosa* in mice model. Administration of HPA3P2 at a dose of 0.5 mg/kg body weight ensured 100% survival rate against MDR *P. aeruginosa* infection. The improved antimicrobial activity is likely due to the altered mechanism of action of HPA3P. Instead of forming pores in the bacterial plasma membrane, HPA3P permeates the phospholipid bilayer and binds to intracellular RNA and DNA. Also, HPA3P2 acts on the LPS layer of outer cellular membrane of Gram-negative bacteria (207). Collectively, these findings indicate that the modification of peptides can enhance their antibacterial activity manifold and can be explored as an effective therapy against MDR strains. Peptide engineering has fueled the discovery of short and potent AMP derivatives, several of which are currently evaluated under clinical trials (208).

Milk is known to harbor inherited antimicrobial activity, owing to natural milk components and active factors (209). Colostrum, having high natural immunoglobulins concentration outlines the initial acquired immunity for the new born in ruminants. These immunoglobulins are secreted in mammary secretions. Milk, therefore, plays an important role in ruminants host defense (195). Besides immunoglobulins, milk contain viable cells, including macrophages and neutrophils, which further regulates secretion of an array of immune-related components, *viz*., antimicrobial proteins and peptides, such as, lactoferrin, defensins and cathelicidins, and cytokines. into milk (210). In an interesting study, a modified peptide, L10 (WFRKQLKW), obtained from bovine lactoferrin by selective homologous substitution of amino acids, displayed potent antibacterial and antifungal activity against ESBL producing Gram-negative bacteria and *Candida* isolates (211). Therefore, milk peptide fractions can serve as therapeutic remedial solution against ESBL producers and MDR fungal strains.

Although AMPs possess potent bactericidal effects and are easily metabolized without adversely effecting the feed quality, few constraints, *viz*., high production cost; safety concerns (many natural AMPs, *viz*., melittin, buthotoxin are toxic to eukaryotic cells); chances of resistance development; poor stability during transportation; easy hydrolysis by proteases in the alimentary canal; and pharmacodynamics, pharmacokinetics, and stability claims supported with only a handful of *in vivo* studies, limit their application as antimicrobial therapy; therefore, a lot of efforts and focus are still needed to categorized them as an effective replacement for antibiotics (79).

## Phage Therapy

Bacteriophages due to their inherent bacterial infection and lysis potential have sought attention of researchers as an antibacterial agent having medical and veterinary application. Phage therapy targets a narrow group of bacteria and prevents dysbiosis normally associated with antibiotic therapy. The autochthonous bacterial flora remains unharmed during phage therapy (212, 213). Phages are obligatory parasites and mainly display two types of life cycle, i.e., lytic and lysogenic (214). In lytic phase, following infection, phage lyses the bacterial cell resulting in cell death. However, during lysogeny, phage DNA gets integrated into the bacterial genome. Lytic phages have attracted attention as potent antimicrobials. The mode of action of lysogenic phages includes adsorption to specific bacterial receptors, followed by DNA injection, redirection of host metabolism, DNA replication, and phage protein synthesis. Later, assembly, packing, and release of phage progeny through cell lysis takes place (215). Bacteriophages have the potential to serve as an alternative to antibiotics in the management of animal disease (216). Lytic bacteriophages are safe and target specific, and can be explored as a potential therapeutic agent against bacterial infections (Figure 4) (217).

Most of the research involving bacteriophage as antimicrobial is mainly foccused on the treatment of enteric and respiratory infections in livestock and poultry (98, 218). It has been observed that intra-muscular inoculation of phages delayed the appearance of *E. coli* in the blood, and enhanced the life span of newly borne calves. Various studies reported that administration of phages either orally or topically in animal models, such as mice, guinea pigs, and livestock are quite effective against different pathogens. Coliphages have been employed for successful management of toxigenic *E*. *coli* and *Salmonella* spp. infections. Lytic bacteriophages have also been explored for preventing *E. coli*-mediated septicemia and meningitis infection in calves (216). In a recent study, Hamza and coworkers (217) described the potential of lytic SA phage against mastitis-causing *S. aureus*. Recently, Sankar (219) also reported that phage therapy could produce beneficial effect against *E. coli-* and *S. aureus*-induced mastitis infection. Earlier, Kwiatek et al. (220) isolated and characterized a bacteriophage having broad-spectrum activity against MRSA isolated from the milk of cows suffering from mastitis. Several earlier reports support the application of phage therapy against bovine mastitis pathogens (221, 222).

Foods of bovine origin had been implicated for possible transmission of *E. coli* O157:H7 to humans (223). Phage mediated *in vivo* studies have been carried out to control the colonization of *E. coli* O157:H7 in cattles (224). Rozema et al. (225) evaluated the efficacy of *E. coli* O157-specific bacteriophages in experimentally inoculated cattle and proposed that regular phage therapy is required to effectively control *E. coli* O157:H7 shedding in feedlot cattle. Later, Rivas et al. (226) screened e11/2 and e4/1c bacteriophages against *E. coli* O157:H7 in cattle and observed that when these two phages were challenged individually against *E. coli* O157:H7 in an *ex vivo* rumen model, cell numbers were significantly reduced. These phages also showed strong potential in reducing *E. coli* O157:H7 on cattle hide (227). Johnson et al. (228) reported preventive effects of phages over multiple pathogens, *viz*., *Campylobacter* spp., *E. coli* O157:H7, and *Salmonella* spp. In 2006, a phage cocktail of six types of pure bacteriophages, designated as LMP-102™ was approved by US-FDA for application as food additives for preventing *Listeria* spp. contamination of meat. Later, another phage product was approved by USDA in 2007 for disinfection of *E. coli* in hidden parts of cattle (79). Phages can also be employed for treatment of viral, fungal, and algal infections in animals (229). The success of phage therapy is limited due to narrow bacterial host range (strict host strain specificity) and due to the requirement of multiple phages for controlling multiple bacterial pathogens. The efficient application of phage therapy requires knowledge about the infectious agent. It is most efficacious when the target pathogen is readily accessible and is present in large numbers (148).

One way to tackle phage associated drawbacks can be the application of purified phage gene products, such as lysins. Endolysins (amidase, endopeptidase, glucosidase and transglycosylase), popularly known as enzybiotics, are mureolytic enzymes generated during the late phage lytic cycle. They target peptidoglycan linkage and lyse bacteria from within, facilitating the release of new phages. Endolysins can quickly kill the susceptible strains and has a wider antibacterial spectrum over phages. Besides, they can also lyse bacteria upon exogenous application (229). Initially, endolysins were shown to be effective in preventing and eliminating bacterial infection by Schuch and coworkers (230). Endolysins isolated from phage φ3626 were also shown to treat *Clostridium* spp. infections (231). Endolysins are specific, highly active, and carries less probability of developing resistance (232). The available literature supports the high possibility of use of phage endolysins for control and treatment of pathogens and infectious disease in dairy animals. However, their antimicrobial application is not yet fully supported with clinical studies. Another bacteriphage mediated strategy can be the application of bacteriophage virion-associated peptidoglycan hydrolases (VAPGHs), those hydrolyzes bacterial peptidoglycan and assists phage entry into the host cell (233). Protein HydH5, a peptidoglycan hydrolase from phage phiIPLA88 showed antagonistic activity against *S. aureus* (234). The antimicrobial spectrum of VAPGHs from Gram-negative bacterial phages is wider over Gram-positive bacterial phages. They are also effective against some antibiotic-resistant pathogens as well, as they exhibit thermal stability, retaining activity at high temperatures (235). Although there are limited studies in context of purified VAPGHs but instance of bacterial resistance has not been reported so far (233). Hence, VAPGHs can prove to be an effective strategy against pathogens exhibiting MDR.

Bacteriophage application in animals is associated with few shortcomings. The biological consequences of phage therapy need to be considered. It is important to avoid temperate phages for therapeutic application, as there is a possibility of transfer of virulence or antibiotic resistance traits from the phage to the host bacterium. Moreover, obligate lytic phages also carry genes of unknown function that could also lead to undesired gene transfer. Other issues related with phage therapy are the lack of clinical trials data, regulatory loopholes, safety (236, 237), stability (215, 236), and difficult to obtain intellectual property rights. Therefore, pharmaceutical companies are little reluctant to invest in phage-based products (238). Consequently, most of the bacteriophage products are still in their research phase (79).

## CRISPR-Cas

Genetic strategies targeting temperate bacteriophage as DNA delivery vehicles, has been proposed to abrogate resistance through enhancing the bacterial sensitivity to antibiotics and selectively killing the resistant strains. Recently, Yosef and coworkers (239) reviewed the possible strategies. Sensitizing genes against target antibiotics are identified and incorporated into bacterial genome through lysogenic properties of temperate phages (Figure 4). Prophages thus generated codes for dominant sensitive genes conferring sensitivity to respective antibiotic(s). The sensitivity genes are also linked to tellurite resistance gene, allowing selection of tellurite-resistant and antibiotic-sensitive strains. Thus, this approach helps in selecting the antibiotic-sensitive strains, eliminating the resistant one. However, this approach suffers from several pitfalls, including the selection of strains sensitive to only selective antibiotics, dependence on prior sensitivity to tellurite, narrow host range of temperate phages, and inability to prevent horizontal gene transfer. In a follow up study, the same group targeted temperate phages to deliver CRISPR-Cas (clustered regularly interspaced short palindromic repeats-Cas) system into host bacteria. The CRISPR-Cas was designed to cleave plasmids carrying antibiotic resistance genes, besides conferring protection to specific lytic phages. This approach linked antibiotic sensitivity to phage protection, therefore, selecting only sensitive strains (240). CRISPR-Cas systems employ CRISPR RNAs to recognize and destroy complementary nucleic acids. These are adaptive immune systems native to bacteria and archaea and can be used for sequence-specific killing of target bacterial strains (241). It works on a selective site and creates a double stranded nick in the DNA, modifying or permanently replacing the target sequence (242). Few other studies have reported the successful application of CRISPR-Cas in managing antibiotic resistance and reversing the selection pressure (243, 244). The CRISPR-Cas system has also been validated in cell culture for its ability to selectively cleave and destroy hepatitis B virus DNA (245). Recently, TATA firm, India in association with Tel Aviv University, Israel has been quoted to have developed a novel technology that restores bacterial sensitivity to antibiotics, reverse their resistance phenotype, alongwith minimizing virulence. The propsed strategy exploits trojan horse strategy, wherein the natural enemies of bacteria are used to inject foreign DNA into bacetrial cell, which results in an attack mechanism killing target bacterium. In contrary, the DNA may help the cells overcome another stress that selectively targets resistant strains. The DNA carries CRISPR, a DNA-editing technology offer that cuts away the antibiotic-resistant genes. Recent developments in CRISPR-Cas genome editing technology and its potential application in food bacteria has been recently reviewed elsewhere (242, 246). CRISPR-Cas technology can have potential application in controlling AMR at dairy farms, through application of developed product, spray, or liquid, etc., in dairy farm envirnonment, dairy personnel hands, etc. However, before the technology can be operational and valued as an alternate to antibiotics, *in vivo* and clinical trials are warranted to standardize it against wide spectra of antibiotic genes, host bacteria, and respective lytic phages (239).

## Immunostimulants

Immunostimulants are the substance that stimulates the immune system by activation of any of its components and enhances the host’s immunity and resistance toward disease in a non-specific manner. They directly enhance innate immune responses through the activation of phagocytes, neutrophils, alternative complement system, and increased lysozyme activity (247, 248). Immunostimulants modulate the immune response against pathogen attack through release of cytokines and cytokine inhibitors; limiting end-organ damage *via* non-specific anti-inflammatory agents (e.g., steroids); and transforming a specific antigen-based response through interferons. In addition, some bacterial substances (β-glucans) and different plant constituents could directly initiate innate defense mechanisms through expression of intracellular gene(s) controlling production of antimicrobial compounds. Presently, the use of immunostimulants as an alternative to the antibiotics is grooming rapidly. Immunostimulants includes wide array of substances, i.e., mineral substances (selenium, zinc); amino acids (arginine, leucine, ubenimex); vitamins (A, E, C); herbals (*Astragalus, Echinacea*); plant polysaccharides (algal polysaccharides, *Astragalus* polysaccharide, chitosan, ganoderan, lentinan, *Polyporus* polysaccharide); microbial preparations (BCG vaccine, cholera toxin B subunit, *Corynebacterium* seedlings, muroetasin, *Mycobacterium phlei*, prodigiosin); bacterial extracts (β-glucan, peptidoglycan, lipopolysaccharide); immunologic adjuvants (aluminum adjuvant, propolis, liposome, Freund’s adjuvant); hormones and hormone-like substances (growth hormone, metallothionein, thymopentin, thymosin); nucleic acid preparations; chemical synthetics (cimetidine, imiquimod, levomisole, pidotimod, polyinosinic acid, sodium houttuyfonate, tilorone, ubenimex); and biological cytokines (interferon, transfer factor, interleukin, immune globulin) (79, 248, 249).

Earlier, Bricknell and Dalmo (250) suggested that the application of immunostimulants as animal feed additives could improve their innate defense and provide resistance against pathogen attack during high-stress periods. Gertsch et al. (251) quoted that the application of plant-based immunostimulants as potential therapeutics is undiscovered and stated that the product acts as a tonic for boosting the immune system without actually specifying its mechanisms. In an interesting study, Li et al. (252) examined the effects of chitosan administration in beef cattle and observed improved immune response and antioxidative function. More interestingly, an immune-stimulant, CpG oligo deoxynucleotides induces a systemic innate immune response for small period that occurs after exposure and it also stimulate B-cell proliferation and expression, production of cytokines, and increased NK cell cytotoxicity (253). Bayers launched Zelnate^®^, an innovative cytosine-phosphate-guanine (CpG) motif-based immunostimulant for animal health. It effectively reduces bovine respiratory disease caused by *Mannheimia haemolytica*. Further research is warranted to describe the specific dosage and efficacy of various immunostimulants. Immunostimulats can be explored to modulate the immune responses which further can act as an adjunct to the antibiotic therapy.

## Cytokines

Cytokines, the intercellular regulatory proteins provide cells with the ability to communicate with one another and orchestrate complex multicellular behavior. They are playing an essential role in normal homeostatic tissue functions but up- or downregulation of their networks are associated with pathological conditions. Thus, cytokines themselves could be considered as indicators of inflammation and a useful parameter in diagnosis of infections (254). Cytokines plays an important role in initiating, maintaining, and regulating the innate immune response and are promising candidates for therapeutic interference in infectious and autoimmune diseases (255). Toll-like receptors (TLRs) also plays an important important role in secretion of cytokines. TLRs are evolutionary conserved surface receptors that recognize structural motifs, *viz*., pathogen associated microbial patterns including lipopolysaccharide, peptidoglycan, flagellin, nucleic acid, etc., of microbial cells. TLRs are predominantly expressed in tissues exposed to external environment and those involved in immue function. Stimulation of TLRs initiates a signaling cascade involving multiple proteins and transcription factors inducing secretion of cytokines directing the adaptive immune response (256). The concentration of cytokines, e.g., TNF-α, IL-6, INF-γ, etc., are usually high in blood serum, milk, uterine washing of cows suffering with subclinical endometritis, mastitis, and other infections and, hence, are proving useful in the diagnosis of these infectious diseases (257).

Cytokines have also been proposed as a therapeutic for bovine mastitis treatment. Application of cytokines alone or as conjunct therapy to antibiotics improves the cure rate of bovine mastitis (258). Recombinant bovine cytokines have also been explored to control and treat bovine mastitis through evoking the host natural defense system. In a study using recombinant cytokines, mammay glands were infused with cytokines (IL-1, IL-2) that led to increased polymorphonuclear cells, with enhanced inducible oxygen radical formation in the milk and thereby effectively preventing *S. aureus* infection (259). The above study indicated that recombinant bovine cytokines can be used to prevent infections in dairy animals; however, more advanced studies are required to be done to accept cytokines as a potential therapeutic and alternative to antibiotics.

## Quorum Quenchers (QQ) or Quorum Sensing Inhibitors (QSI)

Quorum sensing assists bacteria in communication and coordination within themselves and surrounding environment and has been proposed as one of the bacterial mechanism contributing to its pathogenicity (260). Microbial pathogenic behavior is mainly governed through the QS system, comprising of auto-inducers, receptors, and down-stream regulatory proteins, and any pause in the system *via* application of QQ and/or QSI could restrict them (261, 262). Three approaches, *viz*., destruction of auto-inducer through enzymatic cleavage or degradation; disruption of auto-inducer synthesis, and inhibition of ligand/receptor interactions are employed to suppress bacterial QS (Figure 4) (263, 264). QSIs have been classified into peptide (autoinducing peptide homologs), protein QSIs, and non-peptide small molecules. Non-peptide QSIs includes AHLs analogs, l/d-*S*-adenosyl homocysteine and butyryl-*S*-adenosyl-l-methionine; which can interfere with QS signal molecule synthesis or their binding to the receptors (79).

Several veterinary pathogens employ QS for optimizing virulence gene expression and colonization in host. Therefore, any strategy targeting the QS phenomenon among pathogens may help in combating bacterial infections in veterinary medicine, besides addressing resistance. Previously, this strategy has been explored in aquaculture against fish pathogens. Application of AHL analogs reduced the pathogen virulence and associated fish mortality rates (265). Different plants, algae, and fungi have a capability to produce molecules that can inhibit the bacterial QS (266, 267).

Importance of QSIs is reflected from extensive research and increasing number of patents in this field in past few years (268, 269). QSIs appear to be promising under *in vitro* studies; however, all the structural classes of compounds researched and patented have faced some challenges under *in vivo* conditions (270). Although few of the QSI molecules have been tested in preclinical animal models, there clinical application is still un-verified. The QSI, FS3 was screened in a rat model, where it showed good efficacy and synergy with daptomycin (271). Any such agent that disrupts bacterial communication and associated pathogenicity may circumvent the majority of the known resistance mechanisms (270). Direct report of application of QQ and QSIs against MDR strains could not be traced. More concerted efforts are required toward understanding the mechanism and possibilities of large-scale application of QQ or QSIs against infectious and MDR strains.

## Feed Enzymes

Different enzymes are added to animal feed with the target of assisting the digestion and nutrient bioavailability by acting on feed components within the animal’s GIT. There are little chances of enzymatic pre-digestion of the feed substrate during storage (272). Common enzymes used as feed are enlisted in Table 3. These enzymes act as a stimulating factor for the general health and immunity of the livestock which is an important element for reducing the practices of drugs abuse in this field. According to Ravindran and Son (273), a mixture of glycanases and phytase are the most commonly used feed enzymes. For monogastric animals, a range of recombinant synthesized enzymes are commercially available in the form of feed additives (274). However, the advantage of feed enzymes in the form of optimized digestion and enhanced nutrient availability of high-fiber cereal grains and forage is also observed in ruminant livestock (275). Moreover, enzyme like phytase has been reported to have some characteristics effects on mineral (i.e., calcium, phosphorus) digestibility along with the production and secretion of mucin, which influence the organization of intestinal epithelial surface and eventually microbial composition of the gut (276). In addition, these exogenous enzymes could impact on microbial population by providing selective nutritional components to specific group of microbes (277). The direct impact of feed enzymes on innate immunity of animal has also been observed. In a study by Tewoldebrhan and coworkers (278) feeding of β-mannanase enzyme (commercially available as CTCZYME), could reduce the somatic cell counts in milk samples of cows. In light of these findings, it could be deciphered that feed enzymes could be an important factor in controlling the AMR in dairy cattles.

**Table 3:** Enzymes explored in animal feed [modified from Ref. (272)].

Trivial name	Classification	General function
α-Amylase	Carbohydrase	Hydrolyzes starch
β-Amylase		Hydrolyzes starch with production of maltose
Cellulase		Breaks down cellulose
α-Galactosidase		Hydrolyzes oligosaccharides
β-Glucanase		Hydrolyzes β-glucans
β-Glucosidase		Hydrolyzes cellulose with production of glucose
Hemicellulase		Breaks down hemicellulose
Invertase		Hydrolyzes sucrose to glucose and fructose
Lactase		Hydrolyzes lactose to glucose and galactose
β-Mannanase		Hydrolyzes β-mannans
Pectinase		Breaks down pectin
Pullulanase		Hydrolyzes starch
Xylanase		Hydrolyzes xylans

Lipase	Lipase	Hydrolyze tri-glycerides, di-glycerides, and glycerol monoesters

Bromelain	Protease	Hydrolyzes proteins
Ficain		
Papain		
Pepsin		
Protease		
Trypsin		

Catalase	Oxidoreductase	Produces H_2_O and O_2_ from H_2_O_2_
Glucose		Degrades glucose to H_2_O_2_ and gluconic acid

Phytase	Phosphatase	Hydrolyzes phytate

## Nanoparticles (NPs)

Over the last few decades, nanotechnology has evolved as a significant branch of science with wider applications, including those in food, veterinary, and animal sciences, particularly against AMR (279, 280). NPs can be explored as vehicles for delivery of antimicrobial agents (Figure 4). Several reports proposed the potential application of NPs against bovine mastitis and as an alternative to antimicrobial agents against bacteria and fungi (281, 282). Antimicrobial actions of NPs may be through their attachment to the bacterial membrane by electrostatic interaction that may disrupt the integrity of bacterial membrane, alterations in cell wall, blockage of vital enzyme pathways, etc. NPs and their ions induce oxidative stress mediated through generation of reactive oxygen species, which could irreversibly damage bacteria cellular components resulting in death (283, 284).

Antimicrobial activity of nitric oxide and tilmicosin-solid lipid NPs too have been documented against *S*. *aureus* spp. (285). Dehkordi et al. (286) documented the antagonistic activity of silver NPs (AgNPs) against *S. aureus* isolated from subclinical mastitis. Xuefeng et al. (287) reported inhibitory activity of amoxicillin NPs against *E. coli, S. aureus*, and *S. agalactiae*. Berni et al. (288) tested violacein (a powerful anti bactericidal agent) NPs against bovine mastitis and observed high activity of its NPs against *S. aureus*, in comparison to its free form. Kazemi et al. (289) explored synergistic activity of silver NPs (AgNPs) and antibiotics. Co-administration of NPs along with antibiotic inhibited protein translation in *S. aureus* strains. In an interesting study, Kar et al. (290) studied the antibacterial property of AgNPs and capsaicin against MDR and ESBL producing *E. coli* of bovine and poultry origin and postulated that AgNPs and capsaicin could effectively be used to inhibit the growth of MDR-ESBL producing *E. coli*. Alizadeh et al. (291) studied the positive antimicrobial effect of AgNPs against *B. abortus*.

ZnO (Zinc oxide) NPs possess antibacterial, antineoplastic, angiogenic, and wound-healing properties, and has been proposed as a feed additive for mastitis management (279). El-Diasty and coworkers (292) evaluated antifungal potential of ZnO NPs on the growth of dermatophytes and proposed its use as an active ingredient for dermatological applications, whereas Atef et al. (293) studied the potential of iron oxide NPs against the bacterial and fungal skin cattle pathogens. Efficacy of different NPs varies with the type of nanomaterial and its size. Although NPs have shown promise in targeting MDR strains, more detailed understanding of their mechanism of action, associated safety concerns, and the environmental and social implications is warranted.

### Chicken Egg Yolk Antibodies (IgY)

IgY is a major serum immunoglobulin in birds and is available in high concentration from chicken egg yolk. IgY generated by chickens against specific antigens have relatively higher affinity and avidity to antigens and possess high antimicrobial activity (294). These antibodies, besides being safe, economical, specific, and more effective to antibiotics can be targeted against viral or bacterial pathogens, including those having MDR traits (Figure 4). Specific IgY antibodies have been developed against several viral and bacterial pathogens (98). Oral administration of IgY is being currently explored as an alternate strategy to control infectious diseases of gut. Role of IgY in prophylaxis and treatment of rotavirus diarrhea in animal neonates has been recently reviewed in Thu et al. (294). IgY administration has been reported to show promising results in management of several infectious diseases of skin, oral cavity, stomach, intestine, and others. However, being proteinaceous in nature, IgY antibodies are sensitive to GIT stress and may be encapsulated while administering to mammalian gut (294). Recently, avian IgY has also been documented to render protection against dengue (295) and bursal disease virus (296). Available data from *in vivo* and clinical trials clearly points out their possibility to act as an alternate to current day antibiotics. Additionally, IgY antibodies have several merits over current day antibiotics, as reviewed previously by Rahman et al. (297).

## Other Recent Developments

In an interesting study, a small molecule was reported to restore the anmicrobial sensitivity of bacterial strains. Upon co-administration with fluoroquinolones, the small molecule IITR08027 reduced its MIC values against MDR *A. baumannii*. The enhanced sensitivity toward fluoroquinolones was linked to the inhibition of proton gradient and multidrug efflux pump, AbeM. IITR08027 at a concentration of 25 µM decreased the MIC values of several antibiotics to significant levels, extended the post-antibiotic effect, and minimized resistant mutant selection (298). Such new strategies hold promise in minimizing and reversing the phenomenon of multidrug resistance, besides increasing the life of antibiotic.

In a recent update in the field of nanotechnology is the bioseperation of bacteria using bacterial targeted NPs. An interesting study by Lu et al. (299) has presented the use of zinc (II)–bis (dipicoly-lamine) modified NPs for delivering a variety of materials to specific Gram-positive and Gram-negative bacterial population. Authors have effectively produced imageable and magnetically active bacterial constructs using optical dyes or iron oxide colloids containing nanoparticls. Subsequently, labled bacteria were efficiently saperated from the solution with the help of a magnetic column. We could speculate the expension of this technique for *in situ* medical imaging, identification and removal of pathogens, and improved targeted drug dilevery at infection sites in near future.

Against a background of fast emerging resistance to conventional antibiotics, efforts to identify and establish natural alternatives are accelerating and gaining importance. The preceding part attempted to address and propose novel alternative treatment options to current day antibiotics for the welfare of dairy animals. Although a new ray of hope arises with alternative strategies but there is still a huge void between the activity spectrum of antibiotic alternatives and antibiotics itself and regarding their efficacy in disease prevention, growth promotion, and stability. Therefore, intensive efforts from academia, researchers, veterinary doctors, governing bodies, and NGOs are required to propose the control measures through alternative treatments through *in vitro* and *in vivo* experiments, which could take the pressure from current day antibiotics and preserve their efficacy. Overall, from existing updates, alternative strategies are displaying promising and encouraging outcomes. However, it is too early to pronounce that they will entirely replace the current day antibiotics and it is proposed that it will be healthier approach if we will explore them as complementary strategy not as replacement policy.

## Conclusion

Antimicrobial resistance has been identified as a priority research area and mitigation strategies at different fronts are being planned and explored further; however, its impact and spectra is widening at a much faster pace. Alternate strategies suggested herein may not be yet so impactive to completely replace antibiotics as treatment agents, but can be successfully implemented as preventive and management therapy. Along with it, prudent use of antibiotics is quite obligatory to ensure long-term sustainable development of animal husbandry. At the same time, there is need to strengthen the supervision and strict enforcement of laws along with policies pertaining to their usage. Furthermore, there is need to focus on the improvement of animal nutrition and production hygiene. Overall, the dual strategy, i.e., combination of suggested alternative measures along with modest use of antibiotics have promise to pave way for tapping AMR. Moreover, a global, multidisciplinary, long-term approach toward novel diagnostic development and identifying the critical control points is required. Control of AMR should be taken as a “global priority” before it becomes too grim.

## Author Contributions

HP proposed the idea of the review. CS, NR, BS, RG and HP wrote the review draft; JG, PR, and AP helped in improvising the manuscript; NR and CS designed the figures; CS, MC, and HP revised and wrote the final version of the review. The final text has been read and approved by all the authors of the review.

## Conflict of Interest Statement

The authors declare that the research was conducted in the absence of any commercial or financial relationships that could be construed as a potential conflict of interest.
